# Centrosomal P4.1-associated protein (CPAP) positively regulates endocytic vesicular transport and lysosome targeting of EGFR

**DOI:** 10.1038/s41598-021-91818-8

**Published:** 2021-06-16

**Authors:** Radhika Gudi, Viswanathan Palanisamy, Chenthamarakshan Vasu

**Affiliations:** 1 Department of Microbiology and Immunology, Medical University of South Carolina, Charleston, SC 29425 USA; 2 Department of Biochemistry, Medical University of South Carolina, Charleston, SC 29425 USA

**Keywords:** Cell biology, Membrane trafficking, Protein transport

## Abstract

Centrosomal P4.1-associated protein (CPAP) plays a critical role in restricting the centriole length in human cells. Here, we report a novel, positive regulatory influence for CPAP on endocytic vesicular transport (EVT) and lysosome targeting of internalized-cell surface receptor EGFR. We observed that higher CPAP levels cause an increase in the abundance of multi-vesicular body (MVB) and EGFR is detectable in CPAP-overexpression induced puncta. The surface and cellular levels of EGFR are higher under CPAP deficiency and lower under CPAP overexpression. While ligand-engagement induced internalization or routing of EGFR into early endosomes is not influenced by cellular levels of CPAP, we found that targeting of ligand-activated, internalized EGFR to lysosome is impacted by CPAP levels. Transport of ligand-bound EGFR from early endosome to late endosome/MVB and lysosome is diminished in CPAP-depleted cells. Moreover, CPAP depleted cells appear to show a diminished ability to form MVB structures upon EGFR activation. These observations suggest a positive regulatory effect of CPAP on EVT of ligand-bound EGFR-like cell surface receptors to MVB and lysosome. Overall, identification of a non-centriolar function of CPAP in endocytic trafficking provides new insights in understanding the non-canonical cellular functions of CPAP.

## Introduction

Cells can internalize extracellular material, ligands, and surface molecules including receptors by endocytosis. Endocytosis is a fundamental biological process that maintains the cellular, tissue and organismal homeostasis. Internalized molecules traffic through, and are sorted by, a series of tubulovesicular compartments including endocytic vesicles^1^. Vesicular transport pathways play an essential role in the delivery of intra- and extra- cellular cargo, and are critical for cell-to-cell communication^2–6^. Dysregulated cargo delivery pathways can have catastrophic effects and are associated with cancer, neurological diseases, and immunodeficiency^2,7–12^.

Post endocytosis, the cell surface receptors such as epidermal growth factor receptor (EGFR) are routed through different functional stages of endosomes, and can have diverse fates such as: (1) be recycled back to the surface, (2) get targeted for degradation, and/or (3) be released outside the cell in exosomes^13^. Early endosomes (EE) serve as a sorting nexus for, and play a major role in deciding the cellular fate of, endocytosed cargo^1,14^. While the cargo can be recycled back to the surface through recycling endosomes (RE)^15^, regulatory mechanisms exist to ensure that the cargo destined for degradation is routed to lysosomes^16^. Lysosome targeting has two additional steps: (1) the formation of an intermediate endocytic organelle referred to as the multi-vesicular body (MVB), also called as multi-vesicular endosome (MVE) or the late endosome (LE)^17,18^, and (2) the sequential acquisition and activation of GTPases Rab5 and Rab7^7,19,20^. Rab5 to Rab7 conversion is an essential step that marks the maturation of EE to MVBs^21,22^. Each MVB possesses a limiting membrane that encloses several smaller vesicles (referred to as intraluminal vesicles; ILVs)^23,24^. The direct fusion of mature MVBs with lysosomes that contain lipases, lysozyme and other hydrolytic enzymes facilitates the degradation of cargo, potentially terminating deleterious signal transduction by cell surface receptors such as epidermal growth factor receptor (EGFR)^16,25^. MVBs can also be driven towards the plasma membrane to release specific contents as exosomes^26,27^, an essential mode of intra-cellular communication^28–30^. Endocytic vesicular transport (EVT) pathway is known to use microtubules for motor- and Rab- protein regulated movements of endocytic vesicles containing cell surface receptor cargo^31–37^.

In this study, we show a novel positive regulatory influence of CPAP (centrosomal P4.1 associated protein; expressed by CENPJ gene)/SAS-4, a microtubule/tubulin binding essential centriole biogenesis protein^38^ on EVT of internalized epidermal growth factor receptor (EGFR). The centrosome, which consists of a pair of centrioles suspended in a peri‐centriolar material (PCM), is the major microtubule organizing centers (MTOC) of mammalian cells^39^. Several centriole-associated proteins can directly interact with microtubules^40–42^. Among them, CPAP is known to regulate centriolar microtubule growth to produce centrioles of optimum dimension^43^. Importantly, cytoplasmic microtubule function is critical for EVT and targeting of internalized cell surface receptors to lysosome^32,33,35^. To date, CPAP function is primarily attributed to centriole duplication, specifically restricting the centriole length, and ciliogenesis^38,44–47^. Here, by employing gain- and loss-of-function approaches as well as the ligand-bound EGFR intracellular-trafficking model, we assessed the role of CPAP in EVT and lysosome targeting of internalized cell surface receptor cargo. We found that CPAP is required for efficient transport of internalized EGFR to MVB and lysosome for degradation, and our observations suggest that it has a positive regulatory effect on EVT. While the involvement of centrioles and interaction with cytoplasmic microtubules on this function of CPAP needs to be studied, our findings add a new dimension to the potential non-canonical cellular functions of a critical centriole biogenesis protein CPAP.

## Results

### CPAP overexpression causes the formation of punctate structures and increases the abundance of MVBs

**Figure 1:**
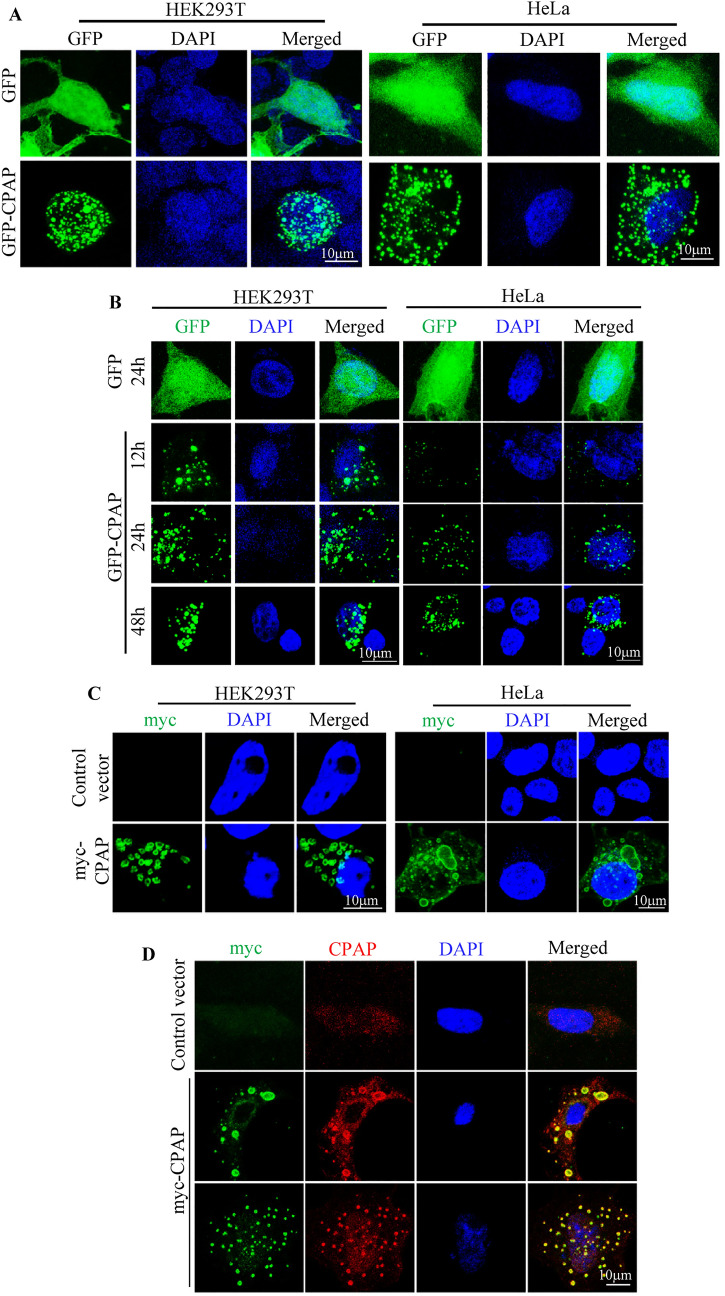
CPAP overexpression causes the formation of vesicular structures. (**A**) HEK293T and HeLa cells were transiently transfected with GFP or GFP-CPAP expression vectors for 24 h and imaged using Zeiss 880 confocal microscope. (**B**) HEK293T and HeLa cells expressing GFP or GFP-CPAP under doxycycline (doxy) -inducible promoter were imaged. (**C**) HEK293T and HeLa cells were transfected with control or myc-CPAP expression vectors for 24 h and subjected to staining using anti-myc antibody and imaging. (**D**) HeLa cells were transfected with control or myc-CPAP expression vectors for 24 h and subjected to staining using anti-myc and anti-CPAP antibodies and imaging. Supplemental Fig. 1 shows IB analysis of various cells used for panels (**A**–**D** for GFP-CPAP and myc-CPAP expression, and IF staining of HEK293T cells that are expressing GFP-CPAP for CPAP. Supplemental Fig. 2 shows that transfection with vector constructs used for exogenous GFP-CPAP and myc-CPAP expression for panels (**A**–**D**) increases the cellular levels of CPAP suggesting overexpression of CPAP. IF staining using anti-myc and anti-CPAP antibodies shows higher binding of antibodies to the outer surface/membrane of the puncta. All results are representative of at least three experiments that produced similar trend in outcomes. All images were processed using ImageJ software version 1.53 (https://imagej.nih.gov/ij/).

The role of CPAP in centriole biogenesis and centrosome function is well established^38,41,48–52^. During our studies to determine the interaction between CPAP and other centriolar proteins, we observed that both HEK293T and HeLa cells transfected with GFP-CPAP or myc-CPAP, and confirmed the expression by IB (Supplemental Fig. 1A–C), formed punctate vesicular structures spontaneously. These punctate structures were observed not only in most cells that were transiently transfected with GFP-CPAP (Fig. 1A), but also in cells that were stably expressing GFP-CPAP under a doxycycline (doxy)-inducible promoter upon doxy treatment (Fig. 1B). Immunofluorescence (IF) microscopy of both HEK293T and HeLa cells that transiently express myc-tagged CPAP (myc-CPAP) also showed similar vesicular structures (Fig. 1C). IF staining for CPAP showed that both myc-CPAP and GFP-CPAP expression induced puncta are CPAP positive (Fig. 1D and Supplemental Fig. 1D). Vesicular structures induced upon GFP-CPAP expression and anti-myc and CPAP antibody staining of myc-CPAP and GFP-CPAP expressing cells appear different, perhaps, due to higher antibody binding to the outer surface of the puncta. Higher cellular levels of CPAP in cells transfected with GFP-CPAP and myc-CPAP, compared to control cells, were confirmed by IB (Supplemental Fig. 2A–C). Overall, these observations suggest that overexpressed CPAP can localize to intracellular vesicular structures.

**Figure 2:**
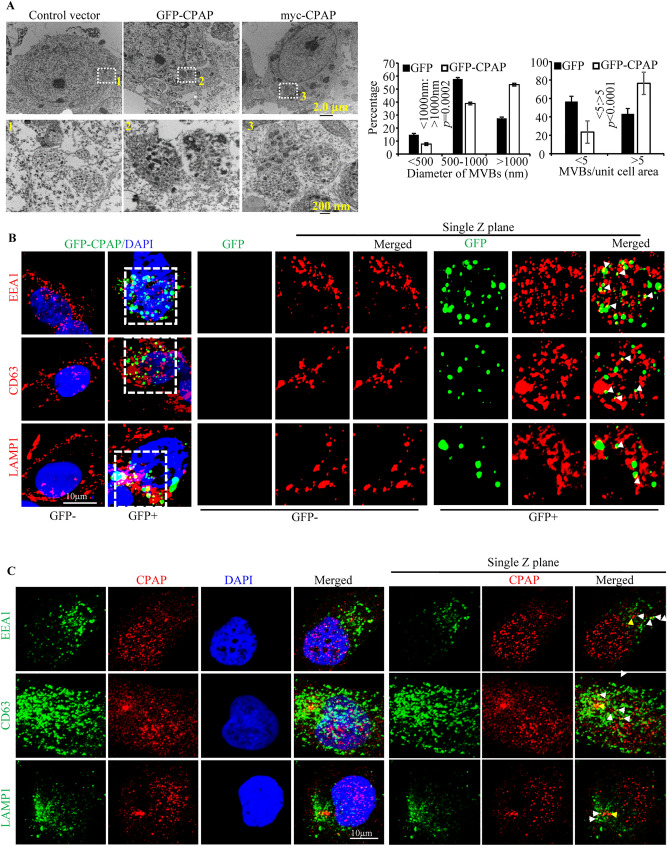
CPAP overexpression causes the formation of MVB-like structures. (**A**) HEK293T cells were transfected with control plasmid, or GFP-CPAP or myc-CPAP expression vectors for 24 h as described for Fig. 1, fixed with paraformaldehyde and processed for TEM, and representative images are shown in upper panels. Numbers of MVB-like structures per unit cell [GFP n = 13; GFP-CPAP n = 43] and diameters of individual MVB like structures [GFP n = 24; GFP-CPAP n = 161] were calculated from three separate experiments for control and GFP-CPAP vector transfected cells and the Mean ± SD values (lower panels) are shown. *p*-value is by chi2 test comparing the ratios of MVBs with < 1000 nm and > 1000 nm sizes or ratios of cells/images with < 5 and > 5 MVBs between control and GFP-CPAP cells. Note: MVB number and size were determined in transfected cell preparations containing a mixture of cells with and without GFP expression in an unbiased manner for both groups. (**B**) HEK293T cells were transfected with GFP-CPAP expression vector (doxy-inducible) for 24 h, treated with doxy to induce protein expression for an additional 24 h, subjected to IF staining for EEA1 (early endosome), CD63 (MVBs), and LAMP1 (lysosomes and late endosome), and images of GFP + and GFP- cells were acquired using Airyscan unit of Zeiss 880 microscope. Maximum projection images of Z-stacks (left panel) and single Z planes (right panel) of relevant representative images from at least 3 independent experiments are shown. (**C**) HeLa cells were stained for endogenous CPAP along with EEA1, CD63 or LAMP1 and imaged using Airyscan unit of Zeiss 880. Maximum Z-projection images (left panel) and single z planes (right panel) of representative images from 3 independent experiments are shown. White arrows show examples of co-localization. Yellow arrows indicate centriole structures. Please note that brightness of red channel was enhanced to visualize low levels of CPAP staining. Supplemental Fig. 4 shows non-enhanced images. Airyscan IF images were initially processed by Zen software (version 2.0) of Zeiss (https://www.zeiss.com/microscopy/us/products/microscope-software/zen.html), and the final maximum projection and single Z-plane images were generated using ImageJ software version 1.53 (https://imagej.nih.gov/ij/). All results are representative of at least three experiments that produced similar trend in outcomes or include cumulative values from three independent experiments. GraphPad Prism software version 9 (https://www.graphpad.com/scientific-software/prism/) was used for determining p-values.

### CPAP overexpression causes increase in the abundance of MVB-like structures

Although CPAP overexpressing cells that were used in previous reports, as shown in Fig. 1, appeared to show similar puncta formation^42,44,45^, the significance of these structures was not investigated. We have performed transmission electron microscopy (TEM) to assess the morphological features of CPAP-overexpressing cells. As shown in Fig. 2A, HEK293T cells transfected with GFP-CPAP and myc-CPAP showed the presence of large, electron dense bodies. These electron dense structures have the characteristics of multi-vesicular bodies (MVB), which are formed when multiple smaller vesicles called intraluminal vesicles (ILVs) are enclosed by a limiting membrane^17,18,25,53^. Interestingly, CPAP overexpression associated MVB-like structures were found to be not only higher in numbers, but also larger in dimensions, than the ones observed in control cells. These observations prompted us to stain GFP-CPAP expressing cells with markers of EE (EEA1), MVB/ LE (CD63) and endolysosome/lysosomes (LAMP1) to assess if the exogenously expressed GFP-CPAP localizes to these vesicular structures with endosomal and lysosomal markers as well as impacts the vesicle morphology. Maximum projection as well as single Z scans obtained using the Airyscan super resolution imager that has a spatial resolution of ~ 100 nm show that a considerable number of GFP + vesicular structures partially overlap with EEA1 + , CD63 + , and LAMP1 + structures (Fig. 2B). These images show that GFP-CPAP structures do overlap with EEA1 + , CD63 + and LAMP1 + structures and are in close proximity. CPAP overexpressing cells also appeared to have larger CD63 + and LAMP + structures compared to control cells. However, IB analysis and DQ-BSA assays showed no major differences in the expression levels of endosomal markers (EEA1 and CD63) and lysosomal marker (LAMP1), as well as the lysosome function in CPAP overexpressing cells compared to control cells (Supplemental Fig. 3). To examine if endogenous CPAP localizes to endocytic vesicular structures, non-transfected HeLa cells were stained for CPAP along with endosome markers. Considering the low levels of endogenous CPAP levels in cells, images were acquired using the AiryScan super resolution module of Zeiss 880 microscope. Maximum projection and single Z-plane AiryScan images show that considerable number of CPAP + structures colocalize with EEA1 + , CD63 + and LAMP1 + structures in cells at steady state (Fig. 2C and Supplemental Fig. 4). Overall, these results suggest that irrespective of the degree of localization of exogenously expressed CPAP to specific functional stages of endosomes at steady state, higher CPAP level contributes to the formation of MVB-like structures.

**Figure 3:**
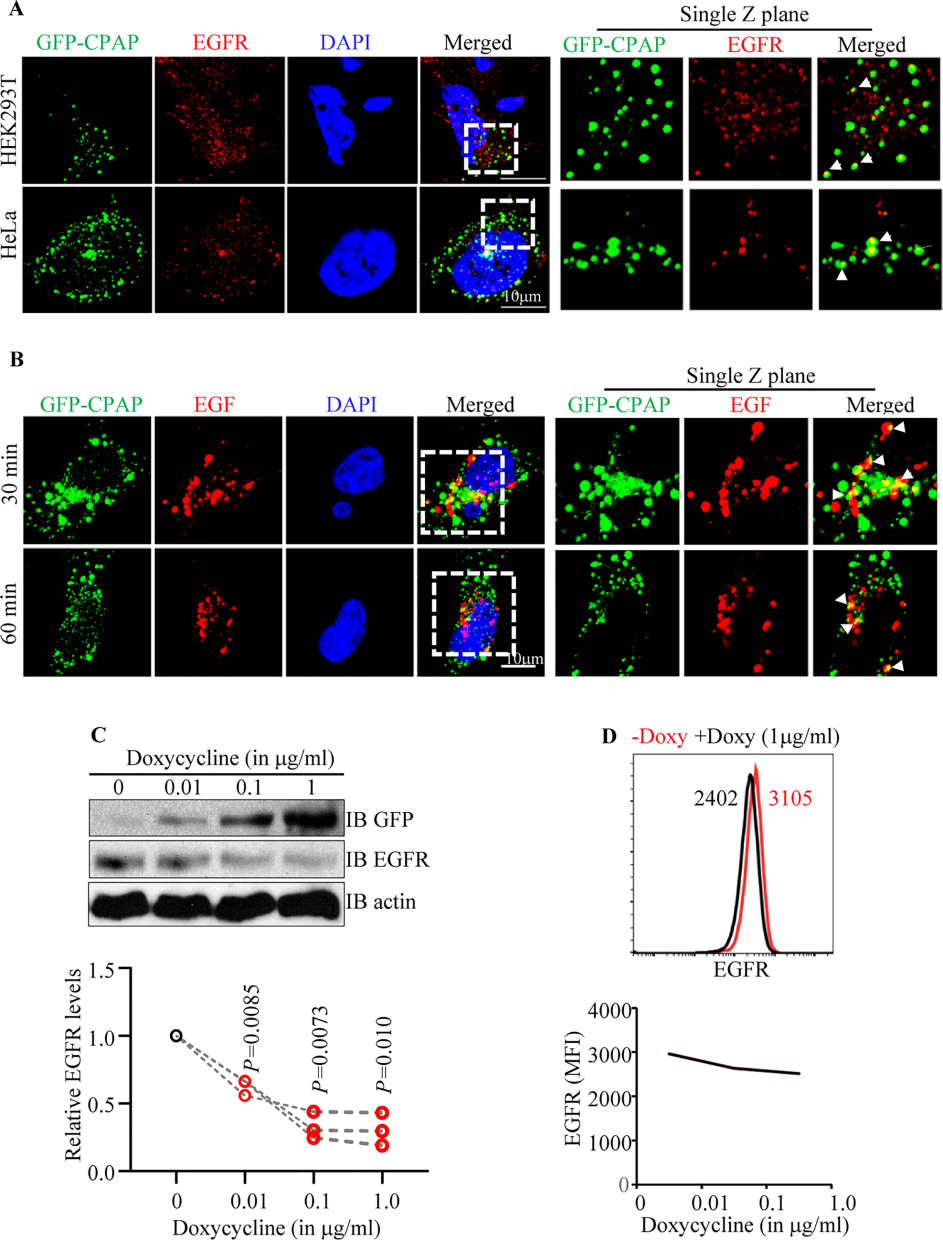
EGFR is detected in CPAP overexpression associated vesicular structures and higher levels of CPAP diminish the cellular levels of EGFR. (**A**) HEK293T and HeLa cells expressing GFP-CPAP under doxy-inducible promoter were stained using anti-EGFR antibody, 24 h after induction of protein expression with doxy (1 μg/ml). Maximum Z-projection images (left panel) and single Z planes (right panel) of representative images from at least 3 independent experiments are shown. White arrows show examples of co-localization. (**B**) GFP-CPAP expressing HeLa cells were treated with Alexa fluor 555-conjugated EGF for different time-points and subjected to confocal microscopy to detect ligand-bound EGFR. Maximum Z-projection (left panel) and single Z-plane (right panel) of representative images from at least three independent experiments are shown. (**C**) HeLa cells stably expressing GFP-CPAP under doxy-inducible promoter were left untreated or treated with varying amounts of doxy for 24 h and subjected to WB to detect GFP-CPAP, EGFR and β-actin. Representative IB images of cell lysates (upper panel) and densitometry analysis (mean of three independent experiments) of EGFR levels at different time-points relative to 0 min time-points are shown (lower panel). *P*-value by paired *t*-test. (**D**) Untreated and Doxy treated cells were surface stained using fluorchrome linked anti-EGFR antibody and examined by FACS. Representative overlay graph with MFI values (upper panel) and mean MFI values of cells (three independent experiments) exposed to different doses of Doxy for 24 h (lower panel) are shown. Original scanned x-ray film images or ChemiDoc imager acquisition images of relevant IB panels are included as Supplemental Fig. 12. All images were processed using ImageJ software version 1.53 (https://imagej.nih.gov/ij/). Flow cytometry data was processed using FlowJo software version 10.0 ( https://www.flowjo.com/solutions/flowjo). All results are representative of at least three experiments that produced similar trend in outcomes or include cumulative values from three independent experiments. GraphPad Prism software version 9 (https://www.graphpad.com/scientific-software/prism/) was used for determining p-values.

**Figure 4:**
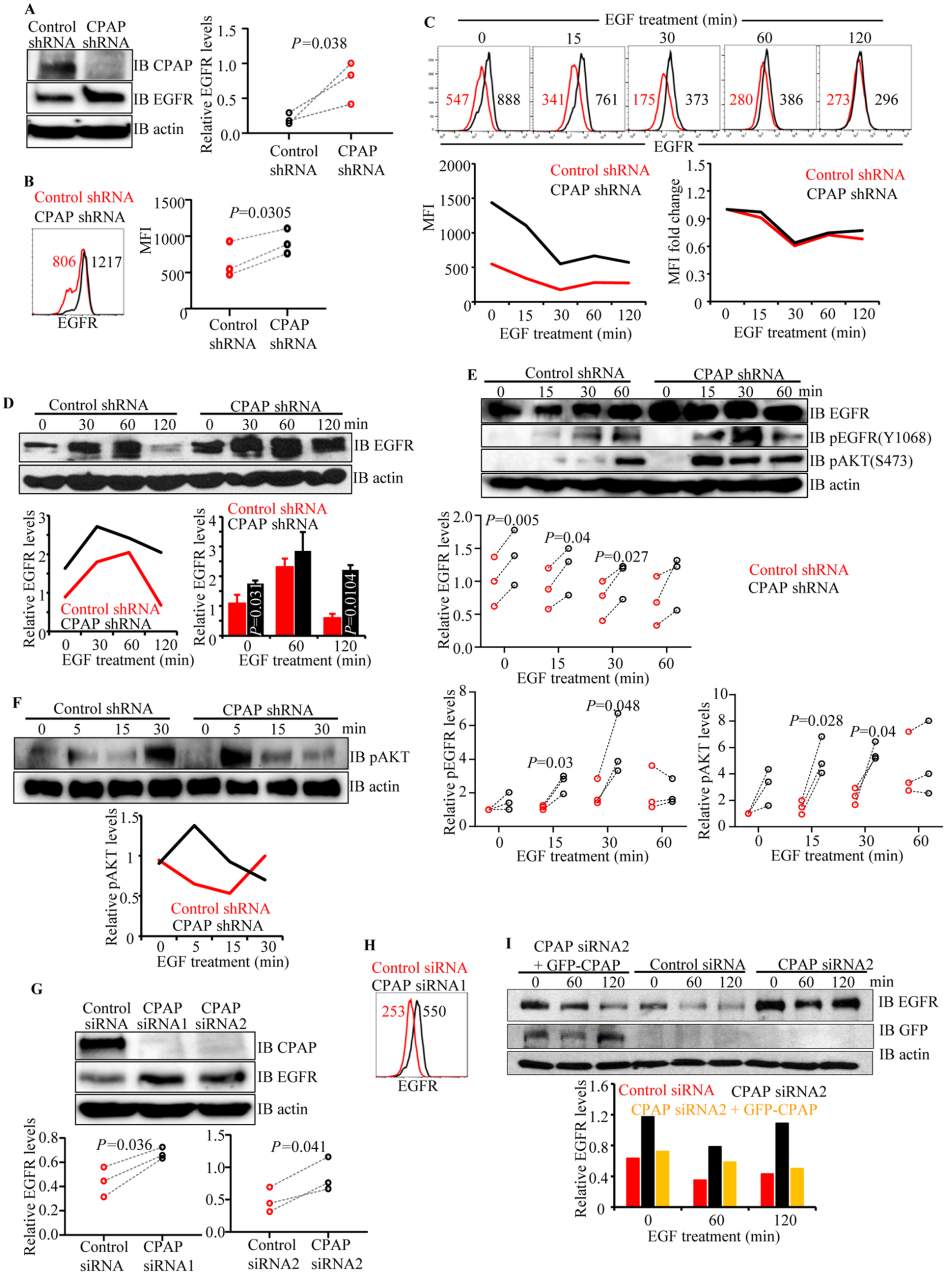
CPAP depletion increases surface and cellular levels of EGFR. HeLa cells stably expressing control-shRNA or CPAP-shRNA were examined for CPAP depletion and EGFR levels by WB (**A**) and surface EGFR levels by FACS (**B**) Panel (**A**) shows representative IB images (left panel) and mean densitometric values (relative to actin; right panel). Panel (**B**) shows representative overlay graph (left panel) and MFI values. (**C**) Cells were subjected to serum starvation overnight, treated with cycloheximide (chx) for 1 h, followed by EGF for 1 h on ice, washed and incubated at 37 °C for indicated durations, and subjected to FACS analysis to detect surface levels of EGFR after staining using anti-EGFR antibody. Representative overlay graphs for each time-point (upper panel), mean MFI values (lower left panel) and EGF treatment induced fold changes in EGFR specific MFI values, relative to 0 min time-point, (lower right panel) are shown. (**D**) Cells stimulated using EGF, as described in panel C, were subjected to IB to detect EGFR and β-actin. Representative IB images (upper panel) and the densitometric quantification of band intensities (lower left panel) and mean ± SD of values (lower right panel) are shown. (**E**) Cells were stimulated using EGF for different durations as described in panel C and subjected to IB to detect total EGFR, phospho-EGFR(Y1068), phospho-AKT(S473) and β-actin. Representative IB images (upper panel) and the densitometric quantification of band intensities (lower panels) are shown. (**F**) Cell were subjected to serum starvation and treated with chx as described for panel C, followed by addition of fresh warm medium containing EGF (no pre-binding of EGF before internalization), incubated for indicated durations at 37 °C, and subjected to IB to detect phospho-AKT (S473). IB images (upper panel) and densitometric values (lower panel) are shown. EGFR, phospho-EGFR and phospho-AKT dynamics under another experimental condition are shown in Supplemental Fig. 6. (**G**) HeLa cells were treated with control-siRNA, CPAP-siRNA1 or CPAP-siRNA2 for 72 h and subjected to IB to detect cellular CPAP, EGFR and β-actin. Representative IB images (upper panel) and mean densitometric values (lower panels) are shown. (**H**) siRNA treated cells were subjected to FACS to detect surface levels of EGFR and representative overlay graph with MFI values is shown. (**I**) siRNA treated cells that exogenously express siRNA-resistant GFP-CPAP under doxy-inducible promoter, were treated with EGF as described for panel D and subjected to IB analysis to detect GFP-CPAP, EGFR and β-actin. GFP-CPAP expressing cells were treated with doxy for 24 h before initiating this assay. Representative IB images (upper panel) and densitometric values (lower panel) are shown. Original scanned x-ray film images or ChemiDoc imager acquisition images of relevant IB panels are included as Supplemental Fig. 12. Flow cytometry data was processed using FlowJo software version 10.0 ( https://www.flowjo.com/solutions/flowjo). All results are representative of at least three experiments that produced similar trend in outcomes or include cumulative values from three independent experiments. *P*-value by paired *t*-test. GraphPad Prism software version 9 (https://www.graphpad.com/scientific-software/prism/) was used for determining *p*-values.

### Cell surface receptor EGFR is detected in CPAP overexpression induced puncta

If CPAP overexpression results in the formation of MVBs and, potentially, constitutive activation of EVT, then cell surface receptors such as EGFR could be continuously targeted to lysosome for degradation in CPAP overexpressing cells. To test this notion, first we examined if EGFR can be found in CPAP overexpression-induced vesicular structures of cells that are grown in complete medium, which may contain low level of EGF of FBS origin. In Fig. 3A, single Z-plane images particularly, show that endogenous EGFR is detectable within or associated with several of the CPAP overexpression-induced spontaneous vesicular structures in both HEK293T and HeLa cells. We then examined if ligand activation induced EGFR (internalized Alexa-fluor 555-linked EGF) can be found in CPAP-overexpression induced vesicular structures. As observed in Fig. 3B, considerable number of internalized, fluorescent EGF-bound EGFR + vesicular structures were associated with GFP-CPAP + puncta. This further supports the notion that CPAP-overexpression associated vesicular structures, at least in part, are perhaps associated with endosomal structures that are known to be involved in the transport of internalized EGFR to lysosome. To examine if the abundance of EGFR is different in CPAP-overexpressing cells, we examined the cellular levels of EGFR in doxy-treated HeLa cells that are stably expressing GFP-CPAP under a doxy-inducible promoter. As observed in Fig. 3C, cellular levels of EGFR, detected by IB, were diminished considerably upon induction of CPAP expression by doxy, in a dose-dependent manner for 24 h. In addition, FACS analysis revealed lower levels of EGFR on the cell surface (Fig. 3D). Overall, these results suggest that CPAP may have a positive regulatory effect in the homeostasis of EGFR-like cell surface receptors. Of note, as reported by us and others before^43,48,49^, exogenous expression of GFP-CPAP caused increase in the centriole size as early as within 24 h of protein induction (Supplemental Fig. 5A).

**Figure 5:**
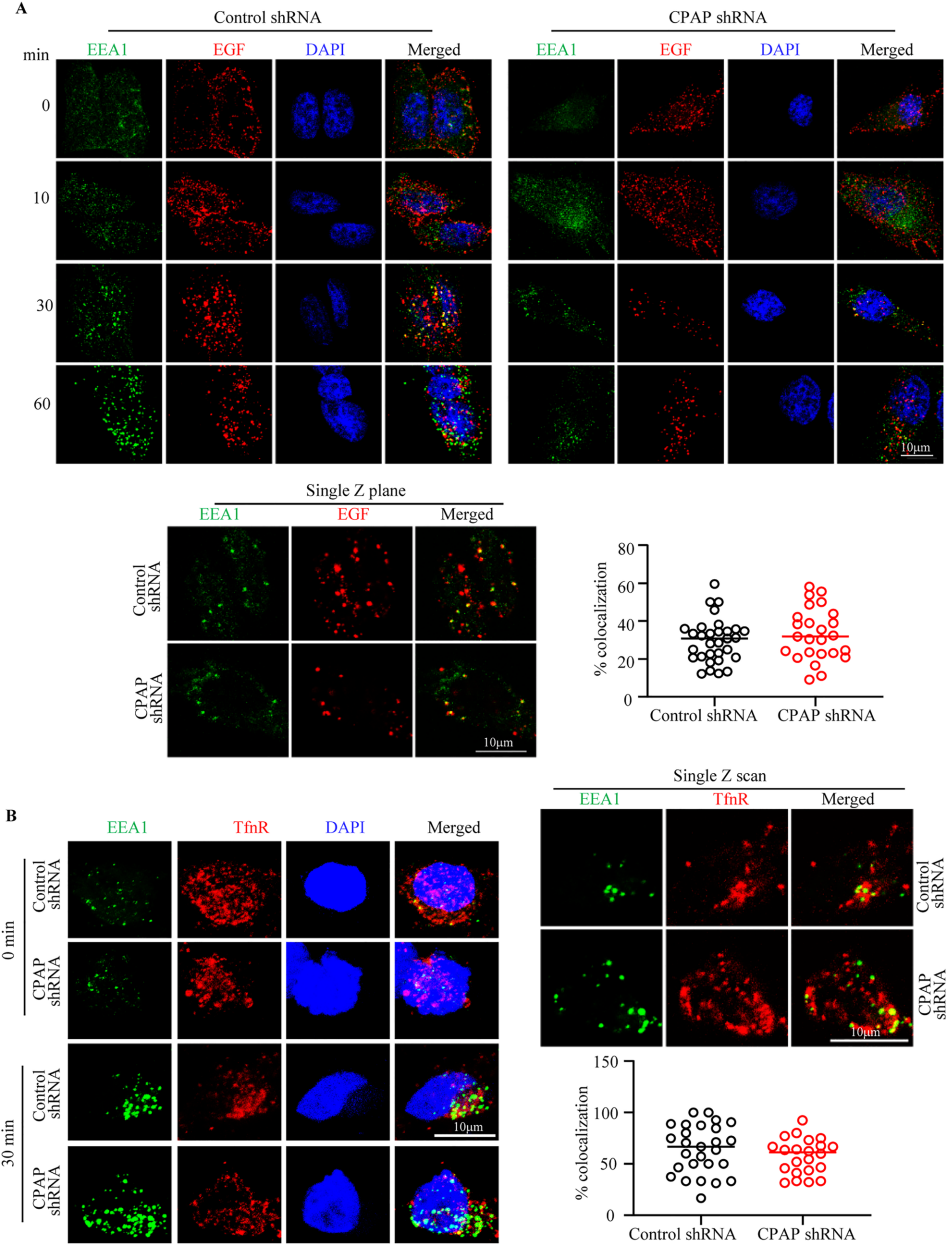
CPAP depletion has no impact on trafficking of cell surface receptor to early endosomes. (**A**) HeLa cells expressing control-shRNA or CPAP-shRNA were incubated with Alexa fluor 555-conjugated EGF ligand and left on ice for 1 h. Cells were washed with serum free media and transferred to 37 °C to initiate receptor internalization. Cells were fixed at indicated time-points, permeabilized and stained for EEA1 to mark early endosomes. Images were acquired as Z-stacks using Zeiss 880 microscope. Maximum projection images of cells harvested at different time-points (left panel), and single Z plane of relevant images (upper right panel) and mean ± SD values (30 min time-point) of percentage EGF + structures that co-localize with EEA1 vesicular structures (lower right panel) from three independent experiments combined are shown. Vesicular structures of a single Z-plane for each cell area were counted. (**B**) HeLa cells expressing control-shRNA or CPAP-shRNA were incubated with holotransferrrin and processed similarly to that for panel A and stained for TfR and EEA1. Maximum projection images of cells harvested at different time-points (left panel), and single Z plane of relevant images (upper right panel) and mean ± SD values (30 min time-point) of percentage TfR + vesicular structures that co-localize with EEA1 structures (lower right panel) from three independent experiments combined are also shown. Vesicular structures of a single Z-plane were counted for each cell area. All images were processed using ImageJ software version 1.53 (https://imagej.nih.gov/ij/). GraphPad Prism software version 9 (https://www.graphpad.com/scientific-software/prism/) was used for determining *P*-values.

### CPAP depletion causes increased cellular levels of total EGFR

Since CPAP overexpression has a negative impact on EGFR levels, we examined if CPAP depletion, which causes defective centriole duplication^43,48,49^ (Supplemental Fig. 5B), affects the surface and cellular levels as well as the internalization and degradation dynamics of EGFR. First, HeLa cells stably expressing control (scrambled) shRNA and CPAP-shRNA (CPAP-depleted) were examined for cellular (Fig. 4A) and surface (Fig. 4B) levels of EGFR by IB and FACS. Relative to control shRNA expressing cells, the cellular and surface levels of this receptor were significantly higher in CPAP-depleted cells. Next, these cells were treated with the ligand, EGF for different durations, under cycloheximide (chx) treatment to block nascent protein synthesis, and the surface and cellular levels of EGFR were determined. Figure 4C shows that the cell surface levels of EGFR are relatively higher in CPAP-depleted cells compared to control cells at all time-points. However, the surface levels of EGFR in both control and CPAP-depleted cells showed similar degrees of decrease after EGF treatment, as indicated by the fold change in overall mean fluorescence intensity (MFI). This suggests that, although the basal surface levels of EGFR are higher in CPAP-depleted cells, it is not due to an altered rate of receptor internalization upon CPAP depletion. IB analysis of EGF treated cells revealed higher cellular levels of EGFR in CPAP depleted cells at most time-points tested (Fig. 4D), suggesting that EGFR degradation pathway is negatively impacted by CPAP depletion. Notably, while majority of the cellular EGFR appears to have degraded in control cells within 120 min post-treatment compared to 0 min time point, CPAP-depleted cells showed the persistence of significant amounts of EGFR at this time-point suggesting its diminished degradation kinetics when CPAP levels are low. To determine if the altered EGFR levels under CPAP depletion translate to the degree of its potential functionality, cellular levels of phosphorylated forms of EGFR and downstream target AKT were determined. As shown in Fig. 4E,F, and Supplemental Fig. 6, higher levels of phospho-EGFR were detected in CPAP depleted cells at relatively earlier time-points compared to that in control cells. Further, relatively higher levels of phospho-AKT were detected in CPAP depleted cells, compared to control cells, especially at early time-points post EGF treatment. These observations suggest that CPAP depletion is associated with higher cellular levels of EGFR as well as the activation of enhanced downstream events upon ligand binding.

**Figure 6:**
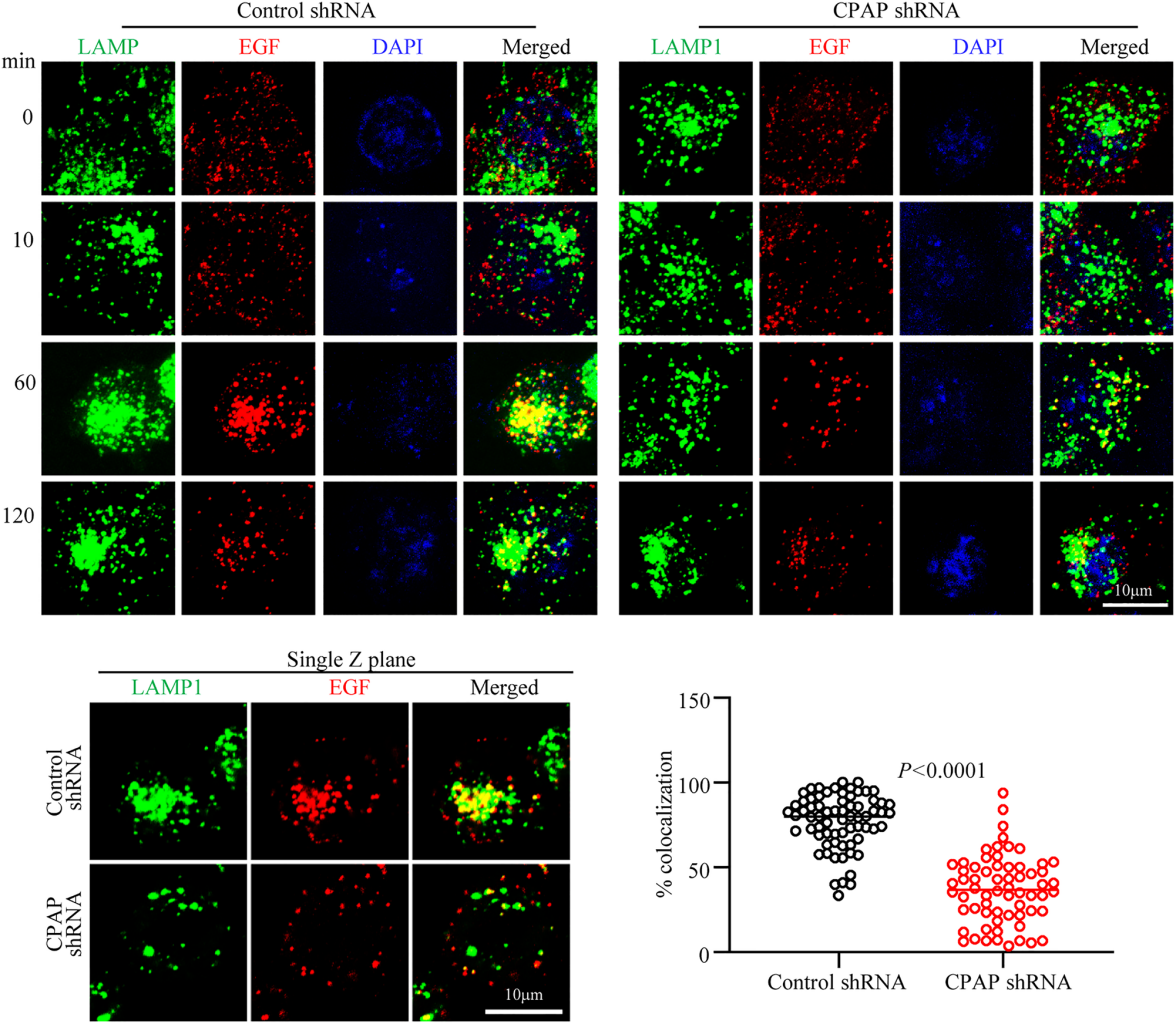
CPAP depletion results in defective targeting of internalized cell surface receptor to the lysosome. HeLa cells expressing control-shRNA or CPAP-shRNA were incubated with Alexa fluor 555-conjugated EGF ligand and left on ice for 1 h. Cells were washed with serum free media and transferred to 37 °C to initiate receptor internalization. Cells were fixed at indicated time-points, permeabilized and stained for LAMP1 to mark lysosomes, and images were acquired as Z-stacks using Zeiss 880 microscope. Maximum projection images of cells harvested at different time-points (left panel), and single Z-plane of relevant images (upper right panel) and percentage of EGF + vesicular structures that co-localize with LAMP1 + structures at 60 min time-point (lower right panel) from four independent experiments combined are shown. Vesicular structures of a single Z-plane were counted for each cell area. Supplemental Fig. 9B shows LAMP1 staining fluorescence intensity of cell areas of multiple experiments. All images were processed using ImageJ software version 1.53 (https://imagej.nih.gov/ij/). *P*-value by unpaired *t*-test. GraphPad Prism software version 9 (https://www.graphpad.com/scientific-software/prism/) was used for determining *P*-values.

To validate the observation that cellular levels of EGFR are negatively impacted by CPAP levels, a similar experiment was conducted after transient depletion of CPAP expression using siRNA which targets different region from shRNA and have been used in earlier reports^46,54^. As observed in Fig. 4G,H, comparable with shRNA expressing cells, HeLa cells treated with CPAP-specific siRNA showed considerably higher basal cellular and surface levels of EGFR. We, then treated HeLa cells with CPAP-specific siRNA and transfected with siRNA-resistant GFP-CPAP cDNA vector^44^ to assess if reintroduction of CPAP function restores the EGFR levels. These cells were treated with EGF as done for Fig. 4D prior to detection of cellular EGFR. Transient depletion of CPAP using siRNA also resulted in higher cellular levels of EGFR, compared to control cells (Fig. 4I). CPAP siRNA treated cells also showed diminished ligand engagement-induced degradation of this receptor, as indicated by little or no reduction in cellular levels after EGF treatment. Reintroduction of CPAP expression appears to restore the ability of ligand treated cells to degrade EGFR, as suggested by its relatively lower cellular levels post-ligand engagement, in comparison with the 0 h time-point. Overall, these observations show defective degradation of internalized EGFR, and its sustained/prolonged function, when CPAP levels are low. These results suggest that CPAP positively contributes to the homeostasis of EGFR and, potentially, other similar cell surface receptors. In fact, Supplemental Fig. 7, shows that CPAP depletion also impacts the cellular levels of TGF-β receptors, which are also known to be similarly targeted to the lysosome for degradation upon ligand engagement^55^.

**Figure 7:**
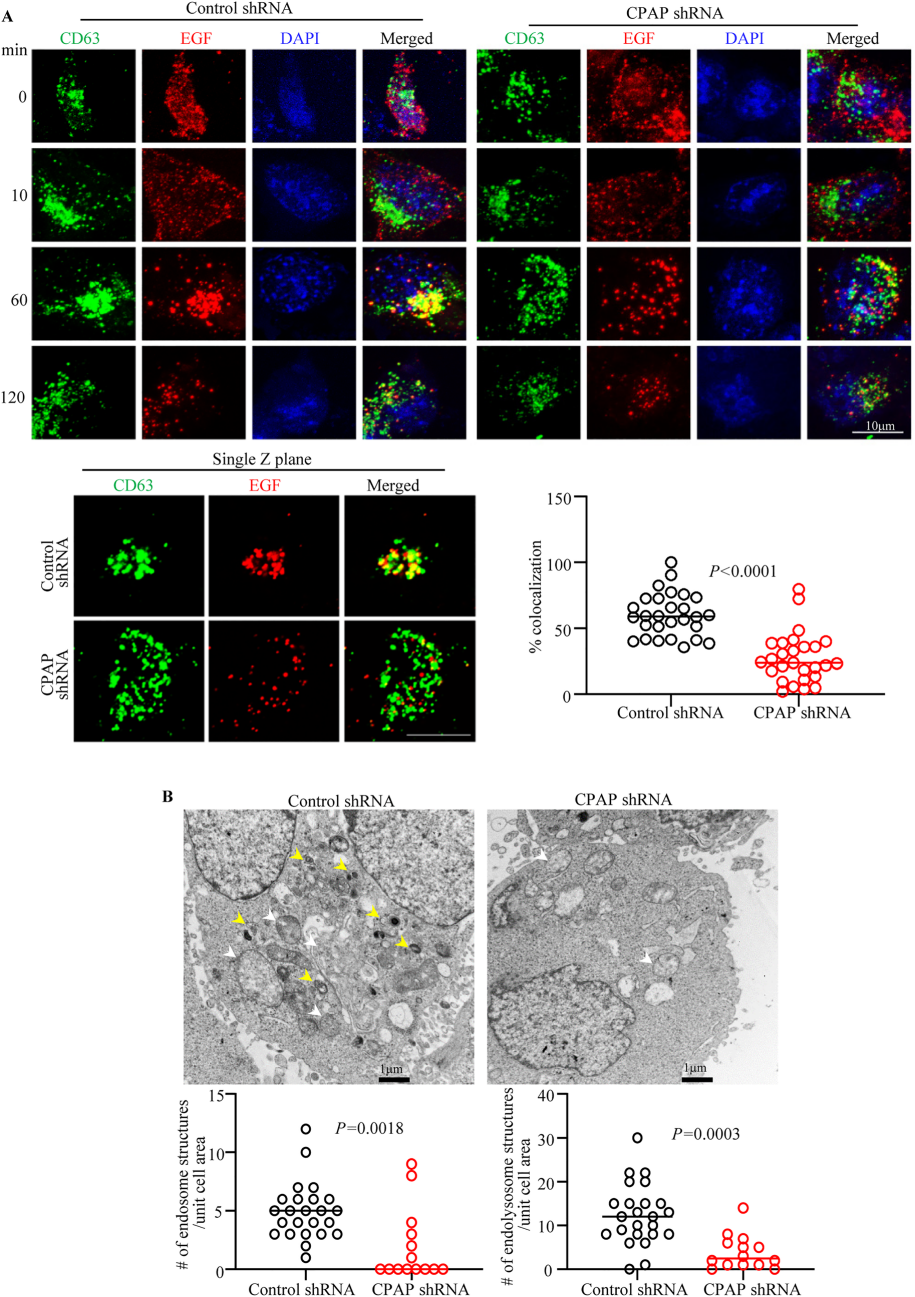
CPAP depletion results in defective trafficking of internalized cell surface receptor to MVB/late endosome. (**A**) HeLa cells expressing control-shRNA or CPAP-shRNA were incubated with Alexa fluor 555-conjugated EGF ligand and left on ice for 1 h. Cells were washed with serum free media and transferred to 37 °C to initiate receptor internalization. Cells were fixed at indicated time-points, permeabilized and stained for CD63 to mark MVB/late endosome. Images were acquired as Z-stacks using Zeiss 880 microscope. Maximum projection images of cells harvested at different time-points (left panel), and single Z-plane of relevant images (upper right panel) and percentage of EGF + vesicular structures that co-localize with CD63 + staining at 60 min time-point (lower right panel) from four independent experiments combined are shown. Vesicular structures of a single Z-plane were counted for each cell area. Supplemental Fig. 9B shows CD63 staining fluorescence intensity of cell areas of multiple experiments. (**B**) HeLa cells expressing control-shRNA or CPAP-shRNA were treated with EGF for 1 h and subjected to TEM analysis, and representative images (top panel) and mean ± SD values (lower panel) of number of endosome structures (indicated by white arrows) and endo-/lysosomes (electron dense bodies; indicated by yellow arrows) from three independent experiments combined are shown. Vesicular/electron dense structures from 1 μm sections were analyzed between the two groups. All IF images were processed using ImageJ software version 1.53 (https://imagej.nih.gov/ij/). *P*-value by unpaired *t*-test. GraphPad Prism software version 9 (https://www.graphpad.com/scientific-software/prism/) was used for determining *P*-values.

### Targeting of cell surface receptors to EE is not affected in CPAP depleted cells

Internalized, ligand-activated receptors are routed to early endosome (EE), following which they are targeted to the lysosome via MVB for degradation or recycled back to the surface^56^. Further, CPAP-depleted cells do not appear to be defective in receptor internalization (Fig. 4C). Hence, we assessed if CPAP has an impact on receptor endocytosis associated events by examining the earliest events of EVT such as EE targeting of ligand bound EGFR, most of which is known to be routed for degradationby the lysosome, in CPAP depleted cells. Control and CPAP depleted HeLa cells were serum starved and treated with chx as described above, and treated with Alexa flour 555-linked EGF on ice for 1 h to allow for uniform surface binding. Cells were then transferred to 37 °C to induce receptor internalization for different durations. These cells were stained for EEA1 and subjected to confocal microscopy to determine the EE localization of ligand bound EGFR. Maximum projection and single Z images, and quantitation of ligand-bound EGFR + puncta localized to EEA1 + vesicles showed that EGFR localizes to EE at a comparable degree in both control and CPAP depleted cells (Fig. 5A). This suggests that CPAP does not influence internalization or transport of receptor cargo to EE. We also examined if the localization of internalized ligand (transferrin; Tfn)-bound transferrin receptor (TfnR), which is primarily recycled back to the cell surface and does not enter the lysosomal degradation pathway^56^, to the EE is affected by CPAP depletion. Control and CPAP depleted cells were treated with Tfn for different durations and subjected to confocal microscopy after staining for TfnR and EEA1. Figure 5B shows that, like EGFR, TfnR is routed to the EE, as indicated by colocalization with EEA1, at comparable degrees in control and CPAP depleted HeLa cells. Further, FACS analysis showed that, unlike the EGFR (Fig. 4), both control and CPAP depleted HeLa cells maintain similar levels of surface TfnR under steady state and ligand treatment (Supplemental Fig. 8) suggesting that this receptor is recycled back to the cell surface at comparable rates in control and CPAP depleted cells. Of note, as shown in Supplemental Fig. 9A, cellular levels of EEA1 were comparable in control and CPAP-depleted cells suggesting that the abundance of EE is also not impacted by CPAP depletion. Overall, in conjunction with the results of Fig. 4 showing the cellular levels and subcellular dynamics of EGFR, these observations suggest that CPAP does not impact the transport of internalized cell surface receptor cargo to the EE or the recycled receptors back to the surface.

### CPAP depletion results in defective trafficking of EGFR to lysosomes

It is well established that ligand engaged cell surface receptors such as EGFR are degraded in the lysosome^16^. Since our results showed diminished degradation and higher cellular and surface levels of EGFR and unaltered EE targeting of internalized receptors in CPAP depleted cells, we examined if targeting of ligand engaged EGFR to the lysosomes is affected in these cells. HeLa cells stably expressing control-shRNA and CPAP-shRNA were treated with Alexa fluor 555-linked EGF on ice, washed and incubated at 37 °C for different time-points and stained for the endolysosome/lysosome marker LAMP1, and subjected to confocal microscopy. As observed in maximum projection images of Fig. 6, control cells showed profound perinuclear clustering of ligand-bound EGFR along with LAMP1 + structures, particularly at 60 min post-ligand treatment. However, compared to these control cells, internalized EGFR as well as LAMP1 + vesicles in CPAP depleted cells showed diminished perinuclear clustering. Single Z scan images and quantification of ligand-bound EGFR-containing structures localized to LAMP1 + vesicles at 60 min time-point particularly, show significantly lower colocalization of ligand-bound EGFR with LAMP1 + structures in CPAP-depleted cells compared to control cells. This suggests an inefficient trafficking of EGFR to the lysosomes when CPAP levels are diminished. Furthermore, lysosome targeting of internalized EGFR appears to be delayed in CPAP depleted cells as indicated by relatively higher perinuclear clustering of EGFR in these cells at later time-point (120 min) compared to the 60 min time-point, as opposed to maximum perinuclear clustering at 60 min time-point in control cells. Experiments using cells that are depleted of CPAP using siRNA also showed similar defective targeting of EGFR to the lysosome (Supplemental Fig. 10). Of note, we found that LAMP1 protein levels, indicating the overall lysosome mass, was relatively higher in CPAP-depleted cells, particularly in HeLa cells (Supplemental Fig. 9A). However, overall LAMP1 IF staining intensities of control and CPAP-depleted cells before and after EGF stimulation appear to be comparable (Supplemental Fig. 9B). More importantly, assessment of DQ-BSA degradation suggests that lysosomal function is also not significantly different, although modestly higher, in CPAP depleted cells compared to control cells (Supplemental Fig. 9C). Overall, these observations indicate that defective transport of internalized EGFR from EE to lysosomes, but not diminished lysosome function, is perhaps the reason for higher total- and phospho- EGFR levels in cells under CPAP deficiency.

### CPAP depletion results in defective trafficking of EGFR to MVB/late endosomes

Late endosomes/MVBs/LE, which are matured from EE, are an important trafficking intermediate which fuse with the lysosomes and form endolysosomes that contain proteases for delivery of degradation of the internalized cargo proteins^57^. Since we observed: (1) higher abundance of MVB in CPAP overexpressing cells (Fig. 2A) and (2) lysosome targeting of EGFR is negatively impacted by CPAP deficiency, we examined if the transport of internalized EGFR from EE to LE/MVB is impacted by CPAP levels. HeLa cells stably expressing control-shRNA and CPAP-shRNA were treated with Alexa fluor 555-linked EGF for different durations and stained for MVB/late endosome marker CD63 by confocal microscopy. Similar to LAMP staining, maximum projection images of CD63 staining (Fig. 7A) showed profound perinuclear clustering of ligand-bound EGFR and CD63 + vesicular structures in control cells, particularly at 60 min post-treatment. However, in CPAP depleted cells, profoundly less perinuclear clustering of ligand-bound EGFR and CD63 + structures were observed at this time point. Single Z scan images and the quantification of ligand-bound EGFR-containing structures localized to CD63 + vesicles, at 60 min time-point particularly, show significantly diminished colocalization of ligand-bound EGFR with MVBs. These results suggest a diminished transport of ligand bound EGFR to MVBs in CPAP-depleted cells. Similar observations were made with CPAP specific siRNA treated cells that were examined for the localization of ligand bound EGFR to CD63 + vesicles (Supplemental Fig. 11). Overall, these results, along with the data presented in Fig. 5 showing slower and diminished perinuclear clustering of ligand-bound EGFR and LAMP1 + and CD63 + structures in CPAP-depleted cells, show that CPAP levels impact the transport of ligand bound EGFR cargo from EE to LE and lysosome, perhaps by affecting MVB formation. In fact, consistent with the lower perinuclear clustering of CD63 + and LAMP1 + structures at 60 min time-point and, potentially due to diminished maturation of endosomes, TEM analysis of EGF-stimulated CPAP-depleted cells showed significantly lower number of electron dense and MVB and endolysosome-like structures at that time-point compared to control cells (Fig. 7B). Of note, IB analysis and quantification of overall IF intensity of CD63 staining of cells showed comparable CD63 levels in CPAP depleted cells and control cells (Supplemental Fig. 9). Nevertheless, our observations suggest that the diminished ability to form mature MVBs/late endosome, as indicated by lower perinuclear clustering of CD63 + structures, in CPAP depleted cells is responsible for the defective transport of EGFR from EE to lysosome and its degradation. Overall, these studies show that CPAP has a positive regulatory effect on the MVB-dependent endosomal transport of cell surface receptor cargo to lysosome for degradation.

## Discussion

Here, we demonstrate a novel positive regulatory effect for CPAP, an essential centriole biogenesis protein^38^, on lysosome targeted EVT of ligand engaged cell surface receptor using EGFR model. CPAP is a microtubule and α-tubulin binding protein and its tubulin-binding function^52,58,59^ is important for its function on the centrioles, especially in restricting the centriole length to < 500 nm^44–46,59^. Overexpression of CPAP can cause aberrant long centrioles of up to 3 µm^44–46^. In addition, CPAP is required for spindle orientation, which defines normal and asymmetric cell divisions^60^. Mutations in the human CENPJ gene have been associated with microcephaly and Seckel syndrome^61–63^. A hypomorphic mouse mutant of the CENPJ gene not only developed microcephaly of the brain but also accumulated centriole duplication defects^64^. Recently, it was also shown that CPAP regulates progenitor divisions and neuronal migration in the cerebral cortex. Centrioles can serve as basal bodies, which template the formation of cilia^65^. More recently, CPAP has been shown to regulate the length of cilia^44,46,47^, perhaps through its centriolar function. Expression of CPAP is cell-cycle regulated^45^ and it also determines the timing of cilium disassembly^66^. In this study, however, we have uncovered, potentially, a non-canonical function of this centriole biogenesis protein in EVT of EGFR-like cell surface receptors and their functional homeostasis.

MVBs serve as critical intermediates towards curbing receptor-mediated signal transduction pathways by sequestering/sorting the cargo of EE into MVBs and routing these proteins to the lysosome for degradation^25^. Our results show that CPAP levels do not have an impact on the internalization or routing of ligand-engaged EGFR into EE. Further, routing for cell surface receptors such as TfnR that are recycled back to the cell membrane from the early endosome also does not appear to be impacted by CPAP. However, our results demonstrate that abundance of MVBs, potentially their generation, and the transport of receptors which are destined for lysosome degradation, are positively impacted by CPAP. This notion has been substantiated by our observation that ligand engaged EGFR colocalization with CD63 + and LAMP + vesicular structures in cells is profoundly diminished and delayed when CPAP is depleted. Perinuclear clustering is the key feature of LE and lysosome when the protein cargo is targeted for lysosomal degradation^67^. Hence the notion that CPAP facilitates endosome maturation is further supported by our microscopy studies revealing defective perinuclear clustering of EGF + vesicles in CPAP-depleted cells as compared to control cells. Importantly, our ultra-structural studies reveal that higher CPAP expression causes increase in the number and size of MVB-like structures and CPAP depletion results in diminished LE/MVB and endolysosome structures. This suggests that, in fact, fewer mature MVBs are generated, as indicated by their smaller size and lack of perinuclear clustering, when CPAP expression is low, leading to a diminished cargo transport from EE to LE, and to the lysosome.

While previous reports were primarily focused on the centriolar localization and function of CPAP, considerable amount of CPAP is also found in the cytoplasmic pool^60^. In fact, cytoplasmic-centrosomal shuttling of this protein has been described as a mechanism of regulating centriole elongation^45,60^. However, a role of this protein in EVT has never been described. Our studies show that overexpressed CPAP is found on the vesicular structures. We also found that internalized ligand-bound EGFR + vesicles, at least in part, are associated with CPAP overexpression induced puncta. However, if the endosome localization of CPAP is required for its positive regulatory effect on vesicular transport function or mature LE/MVB formation needs to be investigated. Based on our observations from single Z-plane images that CPAP-overexpression induced puncta and well as endogenous CPAP co-localizes with EEA1 + , CD63 + and LAMP1 + structures only partially suggest that endosome localization of CPAP may not be necessary for its positive regulatory effect on EVT of cell surface receptors. One potential molecular mechanism could be that CPAP, directly or indirectly through interaction with EVT associated proteins, influences MVB biogenesis and facilitates the lysosomal targeting of the receptor cargo. Other possible mechanism could be through Rab proteins. Rab proteins regulate several steps of the vesicular transport processes to release the cargo to be sorted for degradation^68^. It has been shown that Rab5 dynamically fluctuates on individual EE, linked by vesicle fusion and fission events along with the degradative cargo such as EGFR and concentrates in progressively fewer and larger endosomes^69,70^. This process occurs when endosomes migrate from the cell periphery to the perinuclear region along the MT where Rab5 is rapidly replaced by Rab7. Since CPAP is a microtubule-associated protein, with high affinity to bind tubulin, it is possible that this function is compromised when CPAP levels are low resulting in a collapse in the endocytic vesicle tethering function, defective cargo movement and vesicle maturation along the microtubule filaments. One caveat to this possibility is that CPAP carries a novel microtubule-destabilizing motif that not only inhibits microtubule nucleation from the centrosome but also depolymerizes cytoplasmic microtubules^42^. In fact, it has been shown that overexpression of CPAP, using the constructs used in our study, resulted in depolymerization of cytoplasmic microtubules^59^. Therefore, how microtubule interaction property, and microtubule destabilization upon overexpression, of CPAP are impacting the MVB formation or EVT, and EGFR degradation needs to be investigated.

In conclusion, our studies have identified a novel, non-canonical function for a quintessential centriole biogenesis protein in the vesicular transport of cell surface receptor cargo targeted for degradation. A wide repertoire of canonical centrosome-associated cellular processes including centriole duplication, mitotic progression, spindle orientation, and ciliogenesis have been attributed to CPAP. However, whether this newly identified role of CPAP in EVT is required for these processes or is an independent function remains to be studied. Further, whether the centriole-localization of CPAP and its tubulin/MT-binding ability attributes to this newly discovered effect on vesicular transport remains to be determined. Nevertheless, novel observations described here will pave the way for new studies to determine if and how the fundamental cellular processes such as centriole duplication and endosome maturation are coupled through CPAP. Importantly, these observations could, potentially, also help better explain the molecular mechanisms of aberrant CPAP expression/function-associated microcephaly, centriole duplication, mitotic and spindle positioning errors and ciliopathies.

## Materials and methods

### Cell lines

HEK293T (National Gene Vector Biorepository), HeLa (ATCC) and U2OS (ATCC) cells were used in this study. These cells were cultured in DMEM media supplemented with 10% FBS, sodium pyruvate, sodium bicarbonate, minimum essential amino acids and antibiotics. Transfection of plasmids was performed using calcium phosphate reagent or TransIT 2020 reagent from Mirus Bio LLC while siRNA was transfected using the TransIT siquest reagent from Mirus Bio LLC.

### Plasmids and reagents

GFP-CPAP and myc-CPAP cDNA expression vector^45^ used in this study were kindly provided by Dr. T.K. Tang, University of Taipei, Taiwan. For stable depletion of CPAP, validated lentiviral constructs (pLKO.1) expressing Mission shRNA targeting the following region 5′-GCTAGATTTACTAATGCCA-3′ in CPAP or scrambled shRNA were purchased from Sigma-Aldrich. For generation of stable cells, lentivirus was generated in 293 T cells using accessory plasmids dR8.2 and VSV-G. Target cell line of interest was transduced with virus and selected for shRNA expression by treatment with the drug puromycin (2 μg/ml). RNAi resistant construct for expressing GFP-CPAP and mCherry-CPAP were kindly provided by Dr. Pierre Gonczy, Swiss Institute for Experimental Cancer Research, Switzerland and the CPAP siRNA targeting sequence has been reported earlier^60^.Primary antibodies used in this study: anti-CPAP (Proteintech; cat#11517-1-AP),-GFP (Proteintech; cat#50430-2-AP) -actin (Proteintech; cat# HRP-600008), -EGFR (Santa Cruz Biotech;sc-373746), phospho EGFR (cell signal; cat# 2234; phospho Akt (Proteintech:, 66444-1-Ig); -CD63 (BD Biosciences; cat# 556019), -EEA1 (Bethyl labs; cat# A301-896A),EEA1 (BD biosciences; 610456)LAMP (DSHB; cat#H4B4). Cycloheximide and doxycycline were purchased from Sigma-Aldrich. Unconjugated EGF ligand was purchased from Tonbo biosciences, Alexa fluor-555 conjugated EGF and DQ-BSA-green (Invitrogen), and holotransferrin ligand was from R&D Biosystems. siRNA targeting different regions of CPAP (CPAP-siRNA1: 5′-CCCAATGGAACTCGAAAGGAA-3′ or CPAP-siRNA2: 5′-AGAATTAGCTCGAATAGAA-3′) or scrambled siRNA control (from Dharmacon) were also used.

### Immunofluorescence

Cells grown on coverslips were fixed with 4% paraformaldehyde and permeabilized using 0.1% saponin containing buffer for 30 min. Blocking with 1% BSA as well as primary and secondary antibody dilutions were made in permeabilization buffer and incubations were done at 37 °C. Images were acquired as Z-stacks using either the confocal or super-resolution Airyscan unit of Zeiss 880 confocal microscope using the 63X oil immersion objective with n.a. 1.4 as indicated. Optimal setting as suggested by the software was used to acquire the Z sections.

### Electron microscopy

HEK293T cells transfected with GFP or GFP-CPAP or myc-CPAP constructs for 24 h were fixed with 2.5% glutaraldehyde in sodium-Cacodylate buffer (Ted Pella Inc) for 30 min and processed as described in^40,71^. After dehydration series with alcohol, cells were embedded in Epoxy resin and cured at 60 °C for a couple of days. 70–100 nm thin sections on copper grids were examined and imaged using the JEOL 1210 transmission electron microscope.

### EGFR internalization assay

HeLa cells were grown in serum free conditions overnight and incubated with media containing cycloheximide [CHX] (5 μg/ml) for 1 h at 37 °C. Cells were treated in CHX media with EGF ligand (10 ng/ml) for 1 h on ice. Cells were washed with chilled serum free media and transferred to 37 °C to induce internalization of receptor. For tracking routing of EGF receptor into vesicles by confocal, Alexa Fluor 555 conjugated EGF (250 ng/ml) (Invitrogen) was used. Untagged EGF (10 ng/ml) (Tonbo biosciences) was used for non-imaging experiments. Cell surface EGFR expression was determined by staining cells on ice with anti-EGFR-Alexa 647 (Biolegend) labeled antibody, followed by acquisition using the FACS verse instrument (BD Biosciences). Data was analyzed using the Flowjo software version 10.0 (https://www.flowjo.com/solutions/flowjo).

### Recycling assay

Similar to EGF treatment conditions, HeLa cells were grown in serum free conditions overnight and incubated with media containing CHX (5 μg/ml) for 1 h. Cells were treated in CHX containing media with holotransferrin ligand for 1 h on ice. Cells were washed with serum free media and transferred to 37 °C to induce internalization of receptor.

### Western blot (WB) and immune blot (IB) assays

Cells were lysed on ice, lysates were spun at 14,000 rpm for 20 min, followed by SDS-PAGE, WB and IB. For determining EGFR degradation, cells were collected at indicated time points and lysed using RIPA lysis buffer containing 0.1% SDS and 1% NP-40 detergent. Scanned images of original x-ray films or ChemiDoc images of relevant IB panels are included as Supplemental Fig. 12.

### DQ-BSA assay

Cells were seeded overnight, added DQ-BSA-Green (10 μg/ml) with or without Bafilomycin (100 nM) and cultured for up to 6 h. Cells were washed, fixed and mounted at 0 h or 6 h and imaged for green fluorescence, and the overall green fluorescence intensity per cell area was determined.

### Image quantification and statistical considerations

Prior to image analysis, the .czi files of Airyscan were automatically processed using the Zen software (version 2.0) of Zeiss (https://www.zeiss.com/microscopy/us/products/microscope-software/zen.html). Images are presented as maximum intensity projection or a single Z plane as indicated. Z-stack images were split into single Z planes and red and yellow pixels were quantified to determine the percentage of colocalization between EGF and the endocytic marker. Quantification of colocalization was done manually in the single Z scans. Single Z-plane of each cell area that showed maximum number of endocytic vesicular structures were considered for quantification and image presentation. In EGF trafficking experiments, total number of red (EGF) and red structures localized to green structures (yellow; EGF + endosome marker) were counted per cell area from multiple experiments using the multi-point tool and counter of ImageJ software version 1.53 (https://imagej.nih.gov/ij/) for determining percentage colocalization. Total fluorescence intensity of immunofluorescent cells was determined by calculating the integrated density using ImageJ software. Experiments were repeated several times and all figure panels presented here represent at least three experiments that produced similar trend in outcomes. Cumulative values or values from a representative experiment were used for graphical presentation of data to indicate trend. *P*-values were calculated using GraphPad Prism statistical analysis software version 9 (https://www.graphpad.com/scientific-software/prism/).

## Supplementary Information

Supplementary Information.

## Data Availability

The datasets generated during and/or analyzed during the current study are available from the corresponding author on reasonable request.
