# Supplementary material for: Centrosomal P4.1-associated protein (CPAP) positively regulates endocytic vesicular transport and lysosome targeting of EGFR
Source: Sci Rep. 2021 Jun 16;11:12689. doi: 10.1038/s41598-021-91818-8 (PMC8209166; doi:10.1038/s41598-021-91818-8)
Supplement: Supplementary file 1 — Supplementary Information. [file 41598_2021_91818_MOESM1_ESM.pdf]

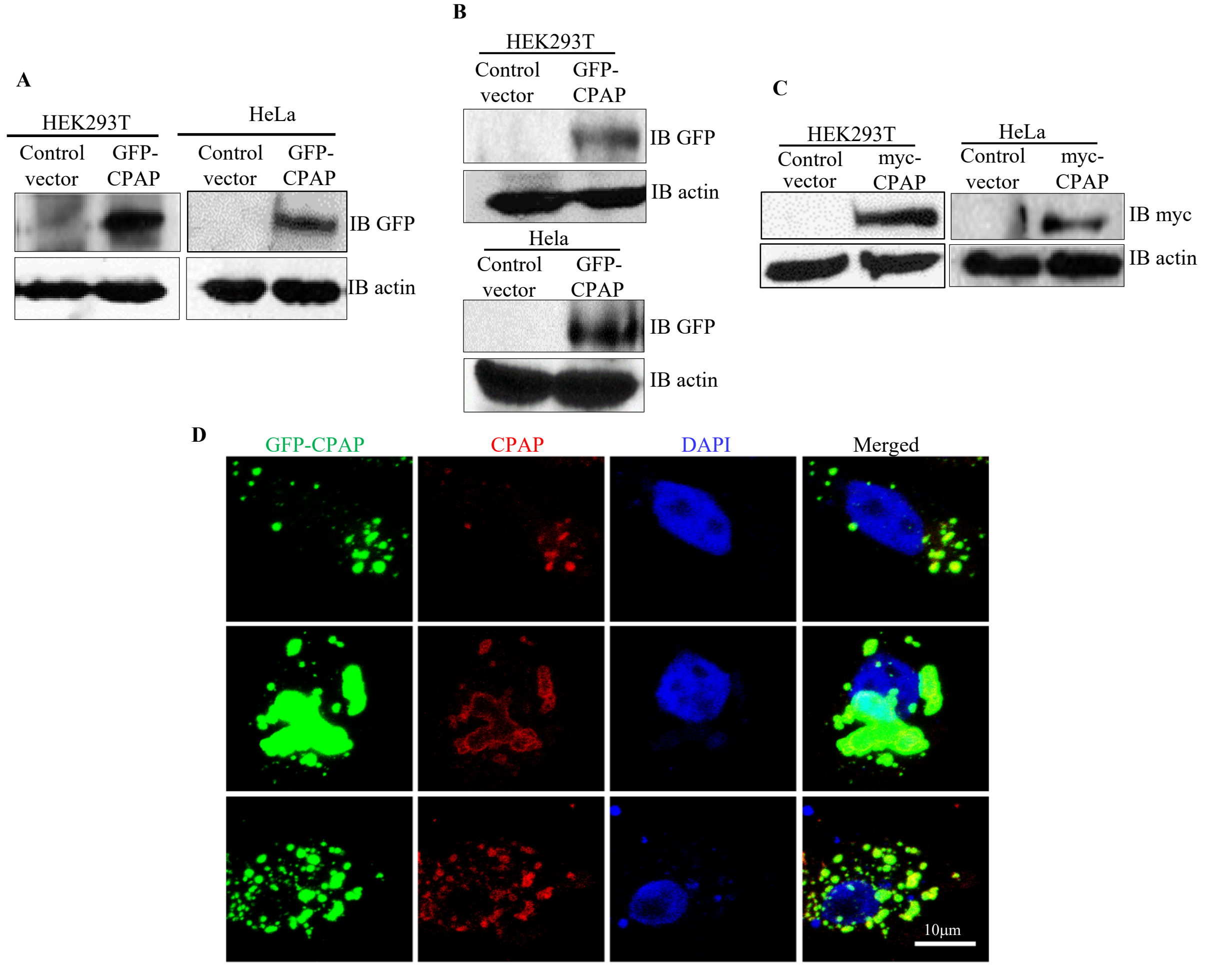

Supplemental Fig. 1: IBs showing exogenous expression of GFP-CPAP and myc-CPAP. A. HEK293T and HeLa cells were transiently transfected with GFP or GFP-CPAP expression vectors for 24h and subjected to IB to detect GFP-CPAP and β-actin. **B.** HEK293T and HeLa cells expressing GFP or GFP-CPAP under doxycycline (doxy) -inducible promoter were harvested 24h post doxy treatment and subjected to IB to detect GFP-CPAP and β-actin. **C.** HEK293T and HeLa cells were transfected with control or myc-CPAP expression vectors for 24h and subjected to IB to detect myc and β-actin. **D.** HEK293T cells were transfected with GFP-CPAP expression vectors for 24h and subjected to staining using anti-CPAP antibody and imaging. Cells with different degree of GFP expression are shown. Higher staining for CPAP was observed on the outer side of the vesicular structures, perhaps due to limited access to the inner part of the puncta. High brightness of GFP gives these vesicular structures puncta appearance. All IF images were processed using ImageJ software version 1.53 (https://imagej.nih.gov/ij/).

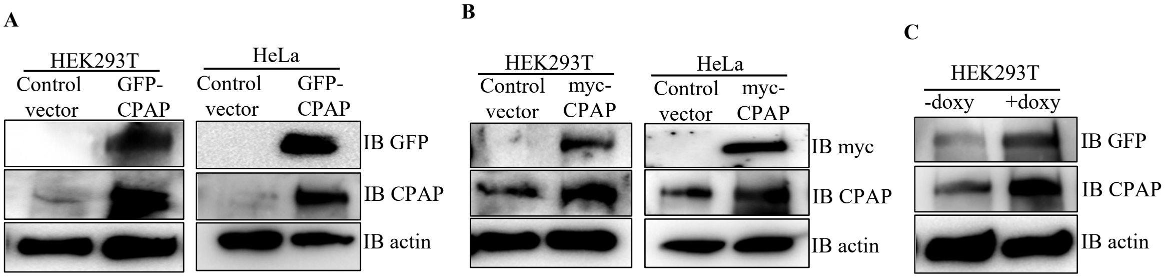

Supplemental Fig. 2: IBs showing exogenous expression of GFP-CPAP and myc-CPAP vectors results in higher levels (overexpression) of CPAP. A. HEK293T and HeLa cells were transiently transfected with control GFP vector or GFP-CPAP expression vectors for 24h and subjected to IB to detect GFP (GFP-CPAP), CPAP, and β-actin. **B.** HEK293T and HeLa cells were transfected with control or myc-CPAP expression vectors for 24h and subjected to IB to detect myc (myc-CPAP), CPAP, and β-actin. **C.** HEK293T cells expressing GFP-CPAP under doxycycline (doxy) -inducible promoter were harvested left untreated (-doxy) or treated with doxy (+doxy) for 24h and subjected to IB to detect GFP (GFP-CPAP), CPAP, and β-actin.

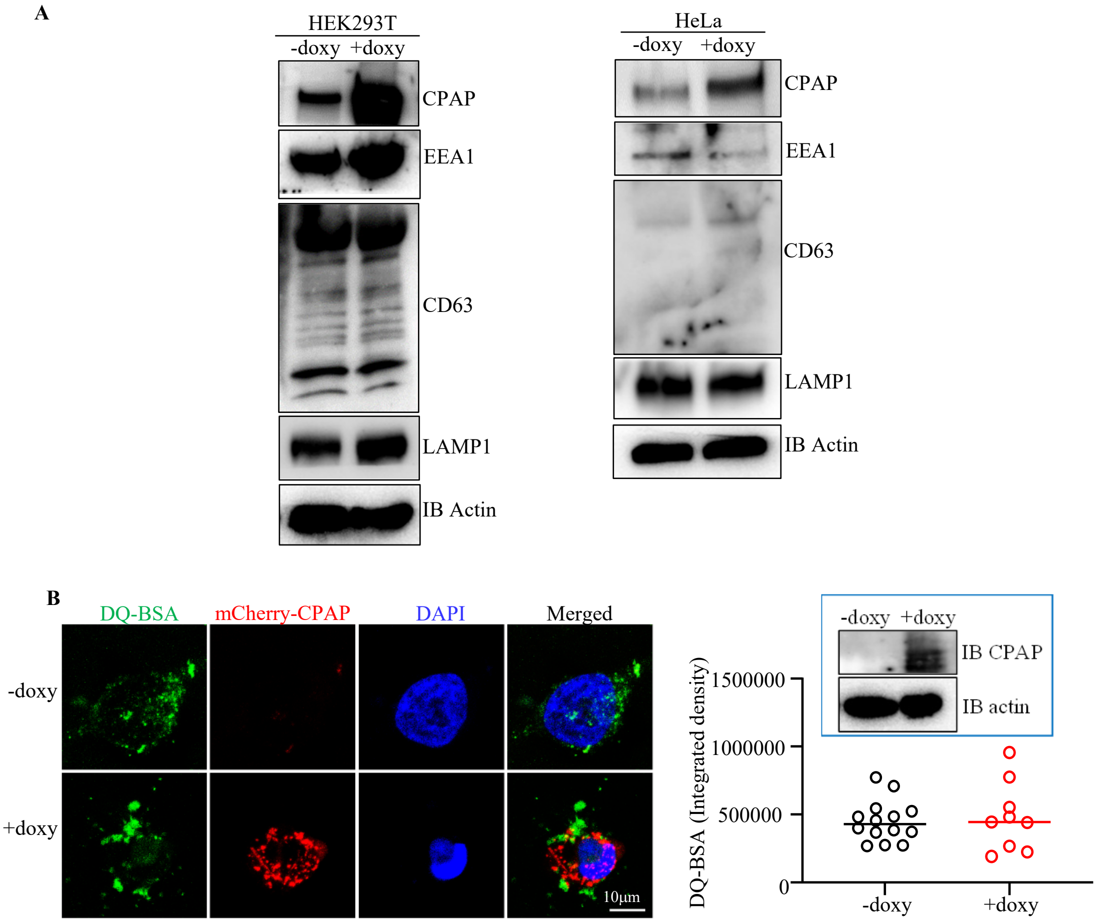

Supplemental Fig. 3: Endosomal/lysosomal marker protein levels and lysosome function in CPAP overexpressing cells. A. HEK293T and HeLa cells expressing GFP-CPAP under doxy -inducible promoter were left untreated or treated with doxy for 24h, and subjected to IB to detect CPAP, EEA1, CD63, LAMP1 and β-actin. **B.** HEK293T cells expressing mCherry-CPAP under doxy -inducible promoter were left untreated or treated with doxy for 24h and incubated with DQ-BSA-green for 6h and subjected to confocal imaging. Representative images (left panel) and fluorescence intensity (right panel) of multiple cells are shown for each group. Right panel also shows IB image of doxy-treated and untreated mCherry-CPAP expressing cells using anti- CPAP and actin antibodies. All IF images were processed using ImageJ software version 1.53 (https://imagej.nih.gov/ij/). GraphPad Prism software version 9 (https://www.graphpad.com/scientific-software/prism/) was used for determining p-values.

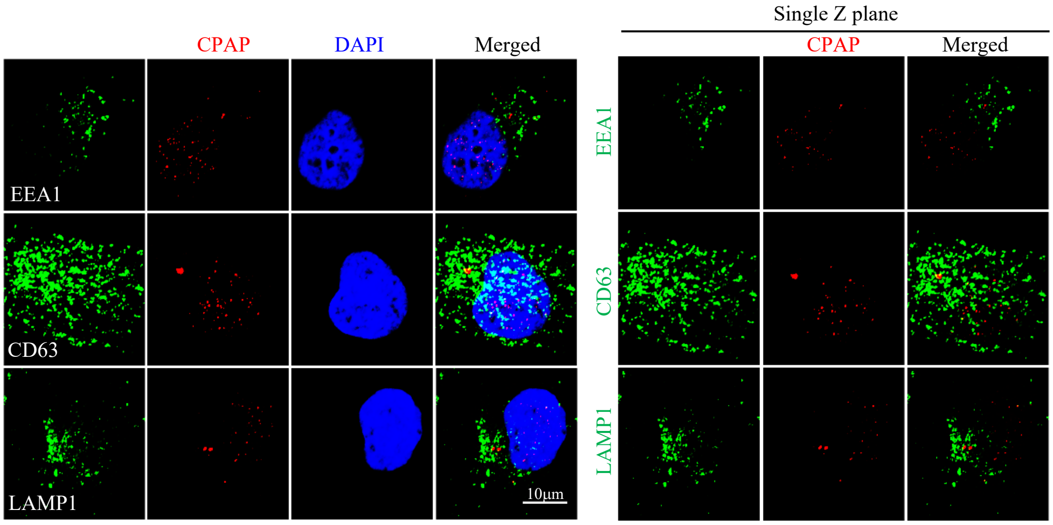

Supplemental Fig. 4: C. Non-transfected HeLa cells were stained for CPAP along with EEA1, CD63 or LAMP1 and imaged using Airyscan unit of Zeiss 880. Maximum Z-projection images **(left panel)** and single z planes **(right panel)** of representative images from 3 independent experiments are shown. Please note that brightness of red channel of these images was enhanced and shown in Fig. 2C. Airyscan IF images were initially processed by Zen software (version 2.0) of Zeiss (https://www.zeiss.com/microscopy/us/products/microscope-software/zen.html), and the final maximum projection and single Z-plane images were generated using ImageJ software version 1.53 (https://imagej.nih.gov/ij/).

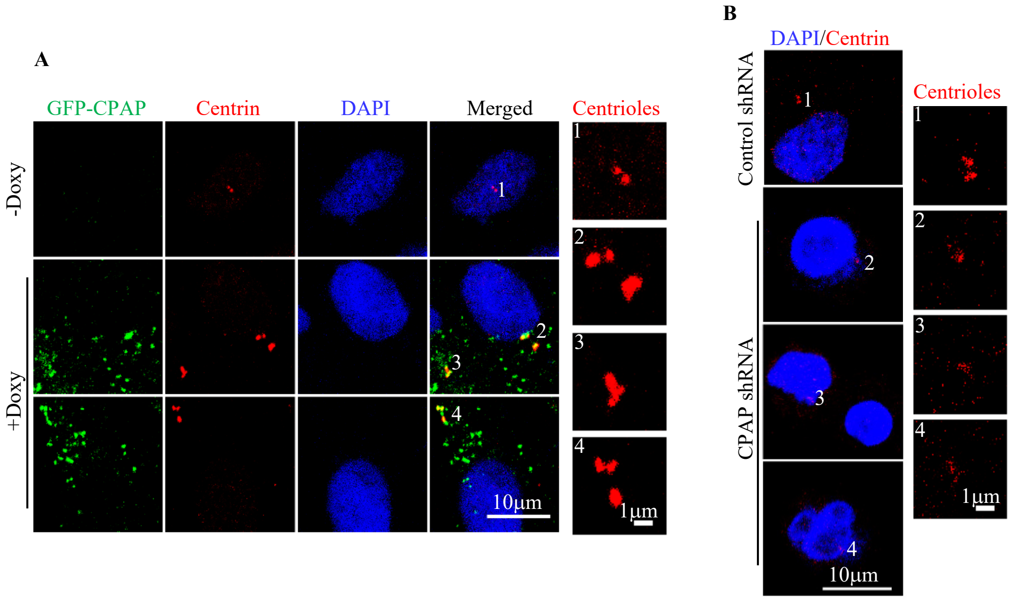

Supplemental Fig. 5: Centriole features in CPAP overexpressing and depleted cells. A. U2OS cells, which are commonly used for centriole elongation studies, expressing GFP-CPAP under doxy -inducible promoter were left untreated or treated with doxy for 24h, and subjected to IF staining for centrin to detect centrioles. **B.** HeLa cells expressing control shRNA and CPAP-shRNA were treated with hydroxyurea overnight to arrest cells in G1/S phase and stained for centrin to mark the centrioles. All IF images were processed using ImageJ software version 1.53 (https://imagej.nih.gov/ij/).

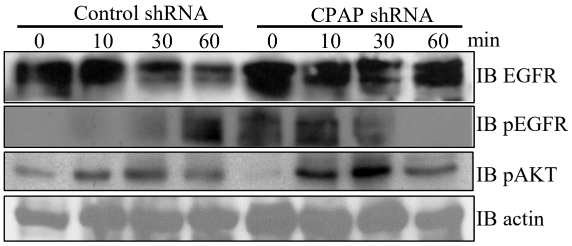

Supplemental Fig. 6: CPAP depletion increases surface and cellular levels of EGFR. HeLa cells expressing control shRNA and CPAP shRNA were subjected to serum starvation overnight, treated with chx for 1h followed by 1h incubation with EGF on ice, changed to fresh warm medium and incubated at 37°C for different durations, and subjected to IB to detect indicated proteins. Representative IB images are shown.

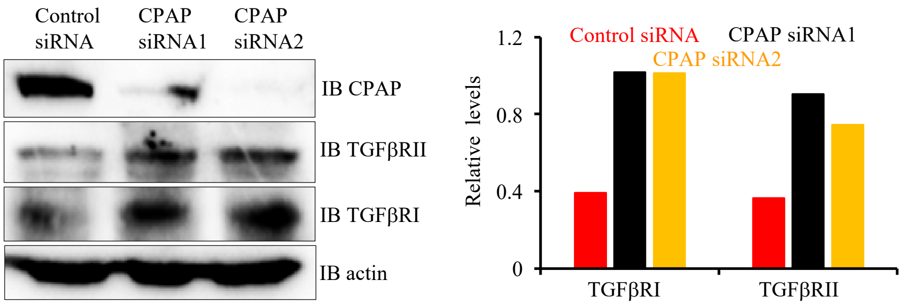

Supplemental Fig. 7: CPAP depletion causes increased cellular levels of TGF-β receptors. HeLa cells were treated with control-siRNA, CPAP-siRNA1 and CPAP-siRNA2 for 72h and subjected to IB to detect CPAP, TGFβRII, TGFβRI and β-actin. IB images (left panel) and densitometry values (right panel) are representative of two independent experiments.

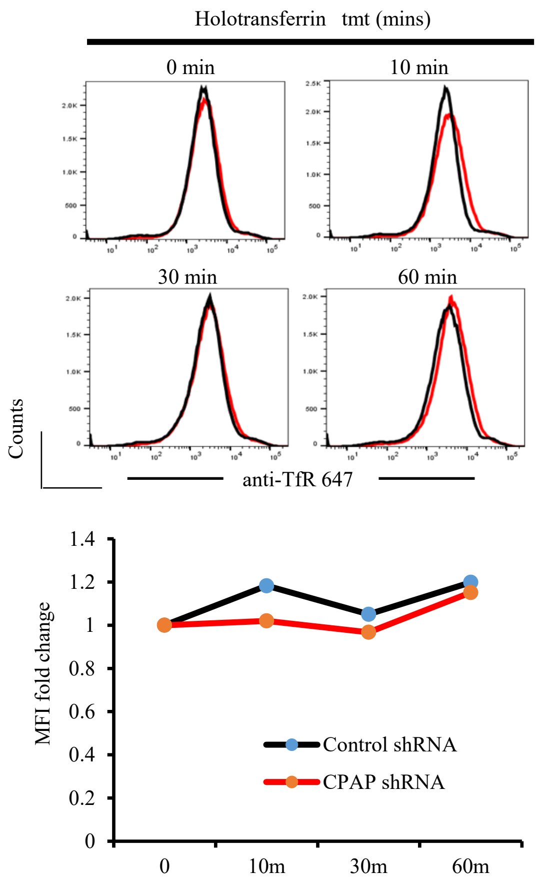

Supplemental Fig. 8: CPAP depletion does not impact surface levels of TfR. HeLa cells stably expressing control-shRNA or CPAP-shRNA were subjected to serum starvation overnight, treated with cycloheximide for 1h and holotransferrin, washed and incubated for indicated durations, and subjected to FACS analysis to detect surface levels of TfR after staining using anti-TfR antibody. Representative overlay graphs for each time-point **(upper panel)** and transferrin treatment induced fold changes in TfR specific MFI values, relative to 0 min time-point, (**lower panel**) are shown. Flow cytometry data was processed using FlowJo software version 10.0 (https://www.flowjo.com/solutions/flowjo).

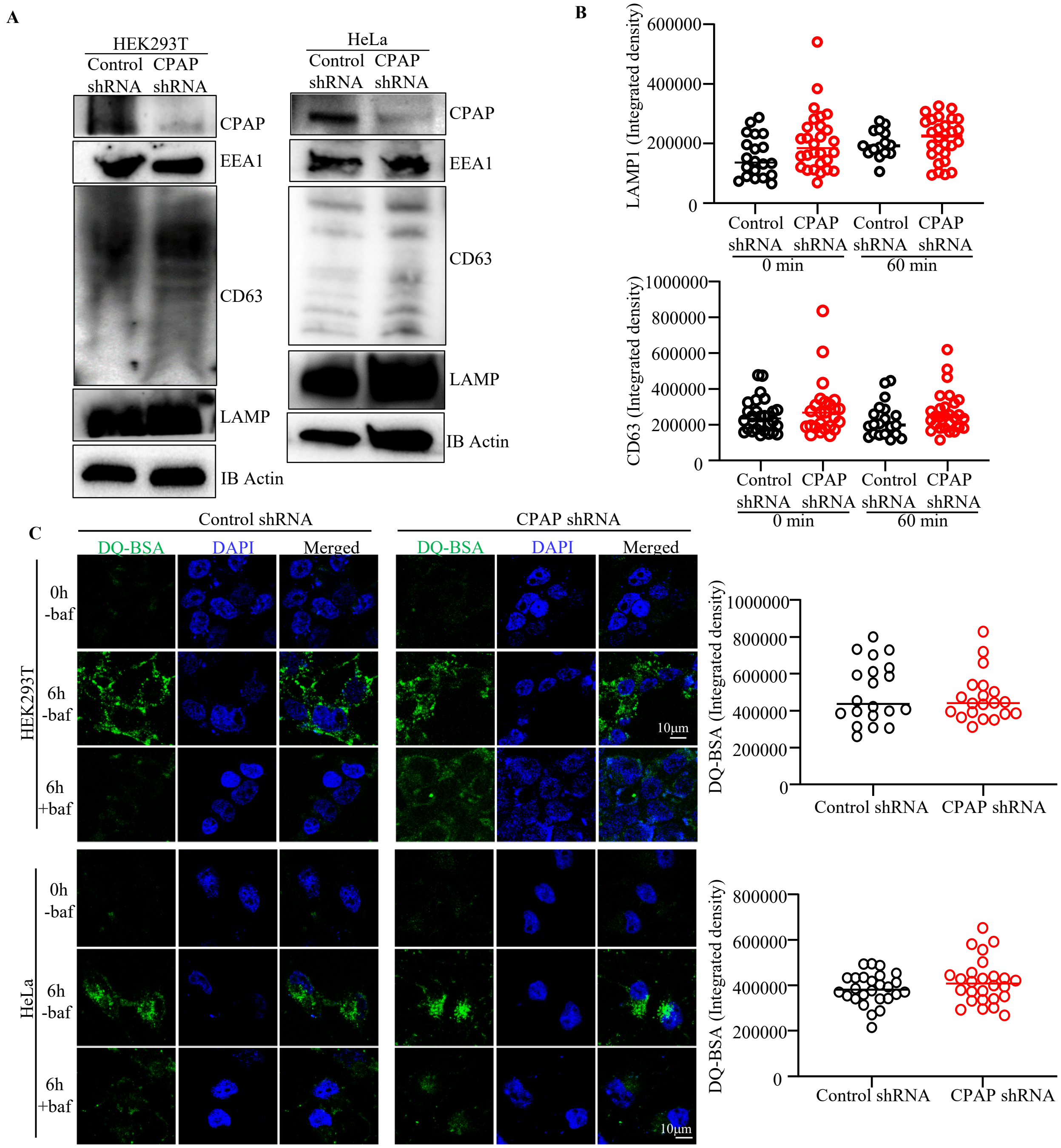

Supplemental Fig. 9: Endosomal/lysosomal marker protein levels and lysosome function in CPAP overexpressing cells. A. HEK293T and HeLa cells expressing control shRNA or CPAP shRNA were subjected to IB to detect CPAP, EEA1, CD63, LAMP1 and β-actin. **B.** LAMP1 and CD63 staining fluorescence intensities of images acquired for Fig. 6 and Fig 7A were quantified and the values of 0 min and 60 min time-points are shown. **C.** HEK293T and HeLa cells expressing control shRNA or CPAP shRNA were incubated with DQ-BSA-green for 6h and subjected to confocal imaging. Bafilomycin (baf) added wells were included as lysosome inhibitor control. Representative images (left panel) and fluorescence intensity (right panel) of multiple cells are shown for each group. Results are representative of 2 independent experiments. All IF images were processed using ImageJ software version 1.53 (https://imagej.nih.gov/ij/).

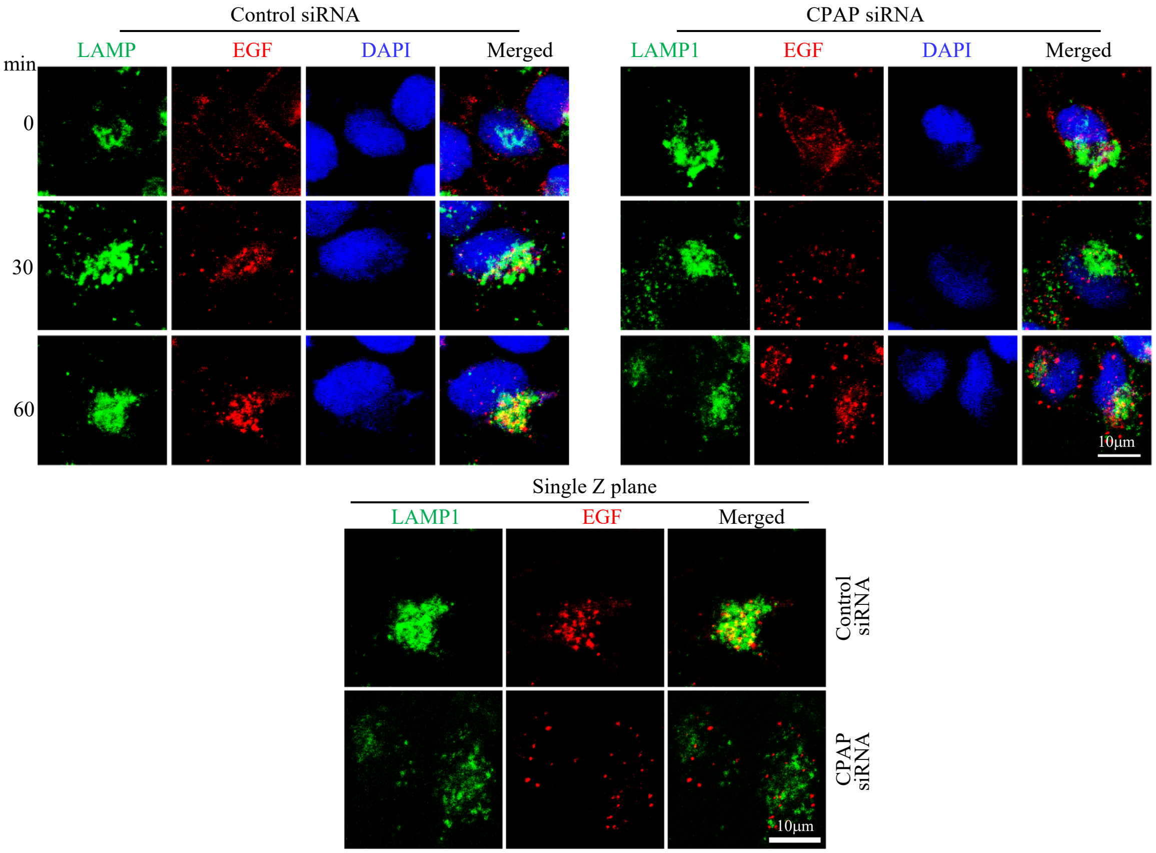

Supplemental Fig. 10: CPAP depletion results in defective targeting of internalized cell surface receptor to the lysosome. HeLa cells were treated with control-siRNA or CPAP-siRNA1 for 72h and incubated with Alexa fluor 555-conjugated EGF ligand and left on ice for 1h. Cells were washed with serum free media and transferred to 37°C to initiate receptor internalization. Cells were fixed at indicated time-points, permeabilized and stained for LAMP1 to mark lysosomes. Images were acquired as Z-stacks using Zeiss 880 and representative maximum projection images (upper panel) and single Z plane of relevant images of 60 min time-point (lower panel) are shown. All IF images were processed using ImageJ software version 1.53 (https://imagej.nih.gov/ij/).

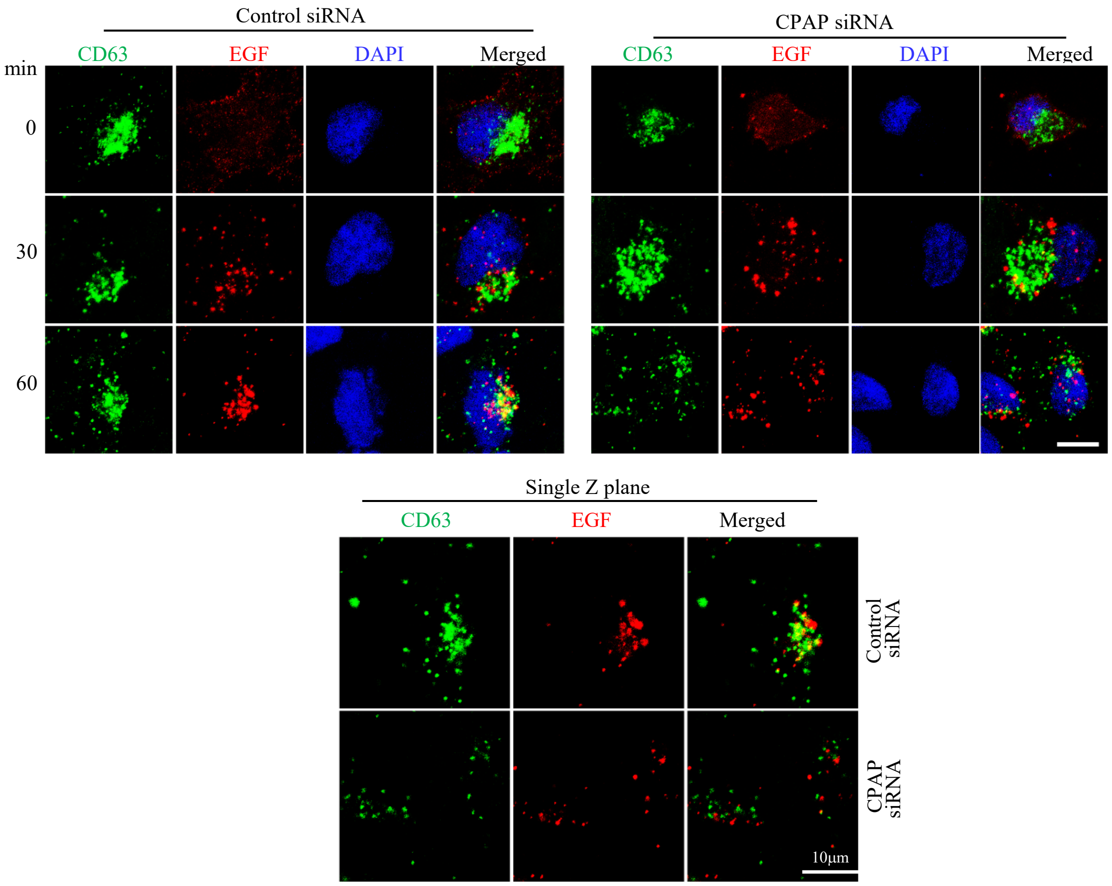

Supplemental Fig. 11: CPAP depletion results in defective trafficking of internalized cell surface receptor to MVB/late endosome. HeLa cells were treated with control-siRNA or CPAP-siRNA1 for 72h and incubated with Alexa fluor 555-conjugated EGF ligand and left on ice for 1h. Cells were washed with serum free media and transferred to 37°C to initiate receptor internalization. Cells were fixed at indicated time-points, permeabilized and stained for CD63 to mark MVB/late endosome. Images were acquired as Z-stacks using Zeiss 880 and representative maximum projection images (upper panel) and single Z plane of relevant images of 60 min time-point (lower left panel) are shown. All IF images were processed using ImageJ software version 1.53 (https://imagej.nih.gov/ij/).

Fig. 3C

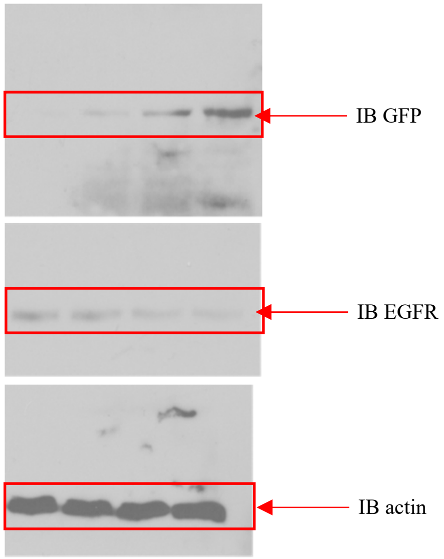

Supplemental Fig. 12: Original IB (scanned x-ray film or ChemiDoc chemiluminesence imager) images. Please note that, for many of the IBs, original WB membranes were cut based on the molecular weight markings before probing using different antibodies. Hence, many comparisons were done using data generated using the same blot, but no parallel blots.

Fig. 4A

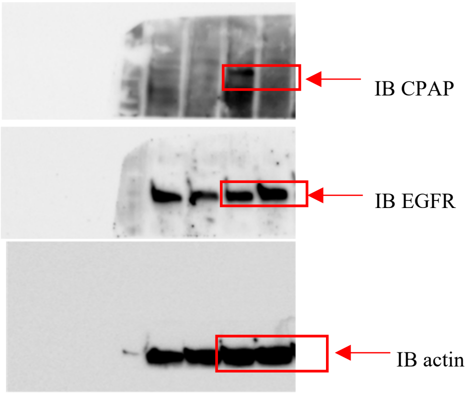

Fig. 4D

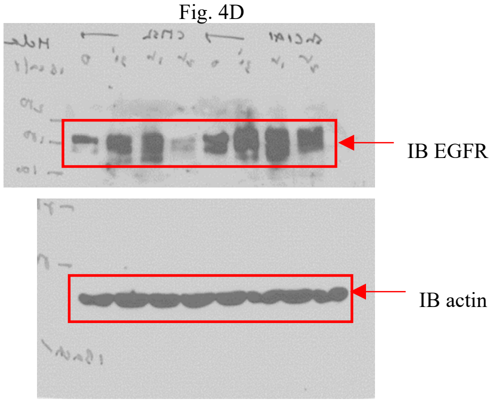

Fig. 4E

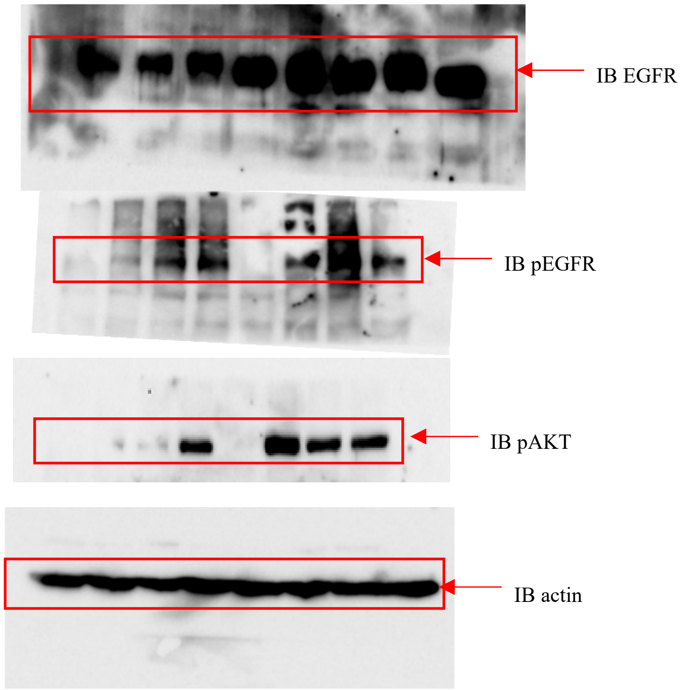

Fig. 4F

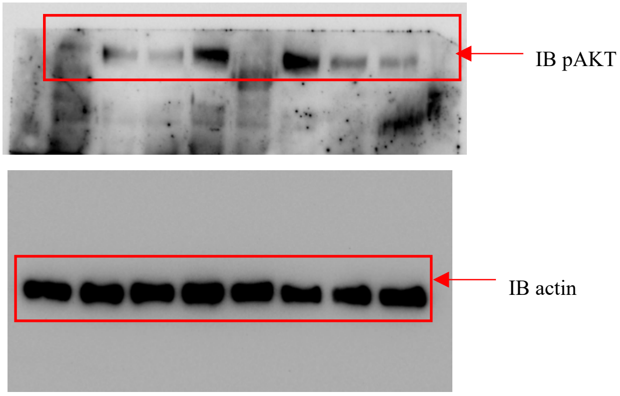

Fig. 4G

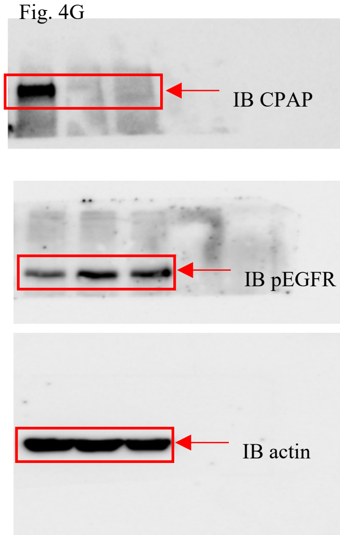

Fig. 4I

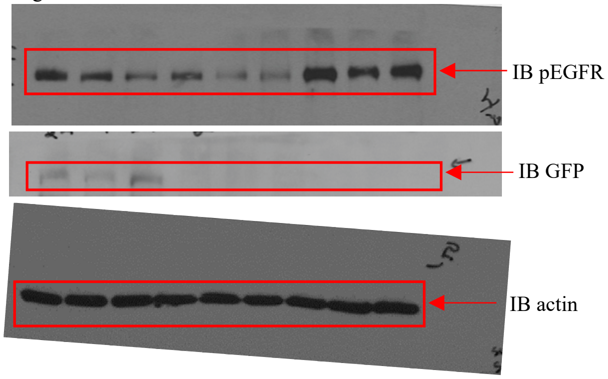

Continue supplemental Fig. 12: Original IB (scanned x-ray film or ChemiDoc chemiluminesence imager) images. Please note that, for many of the IBs, original WB membranes were cut based on the molecular weight markings before probing using different antibodies. Hence, many comparisons were done using data generated using the same blot, but no parallel blots.

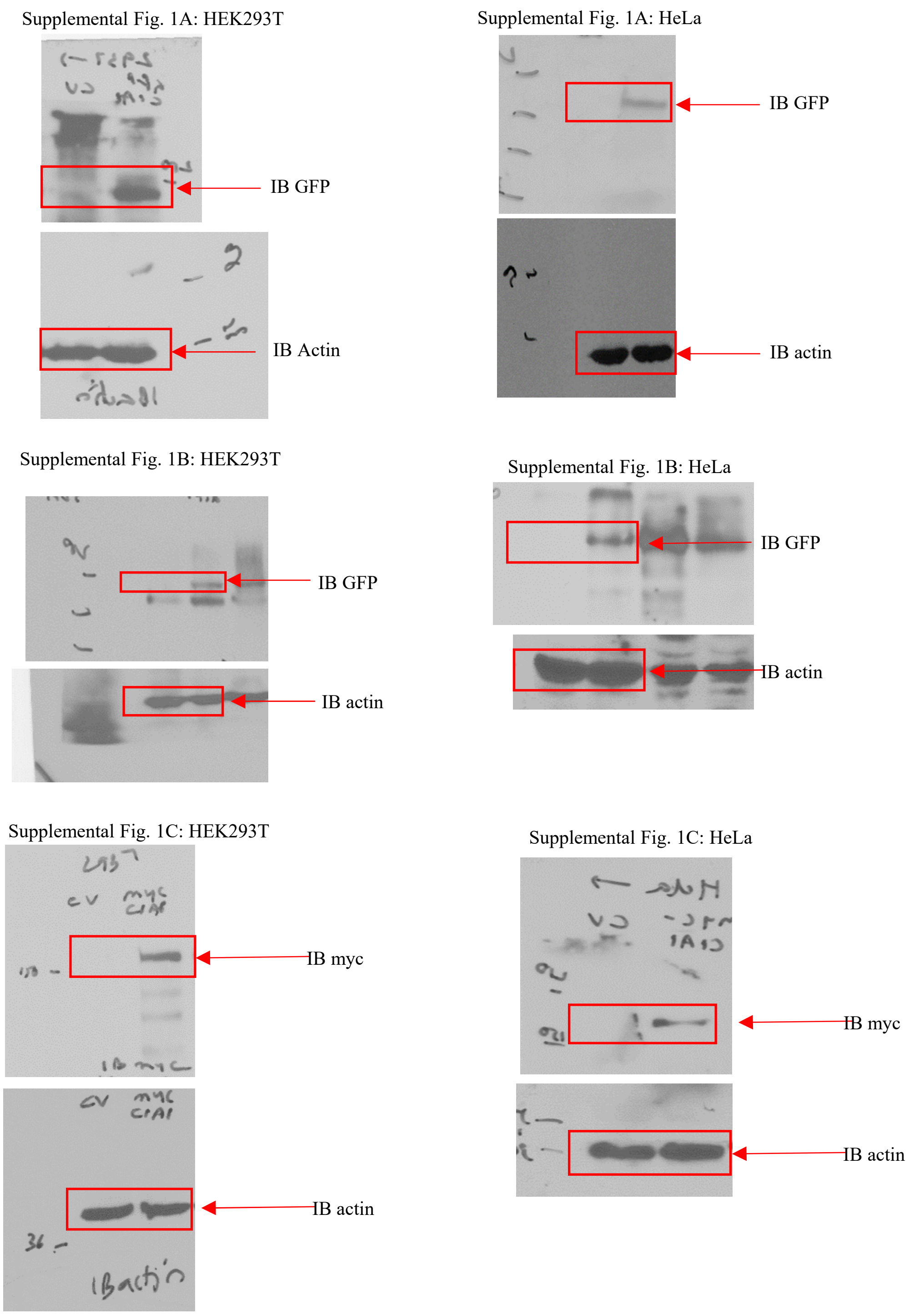

Continue Supplemental Fig. 12: Original IB (scanned x-ray film or ChemiDoc chemiluminesence imager) images. Please note that, for many of the IBs, original WB membranes were cut based on the molecular weight markings before probing using different antibodies. Hence, many comparisons were done using data generated using the same blot, but no parallel blots.

Supplemental Fig. 2A

HEK293T

HeLa

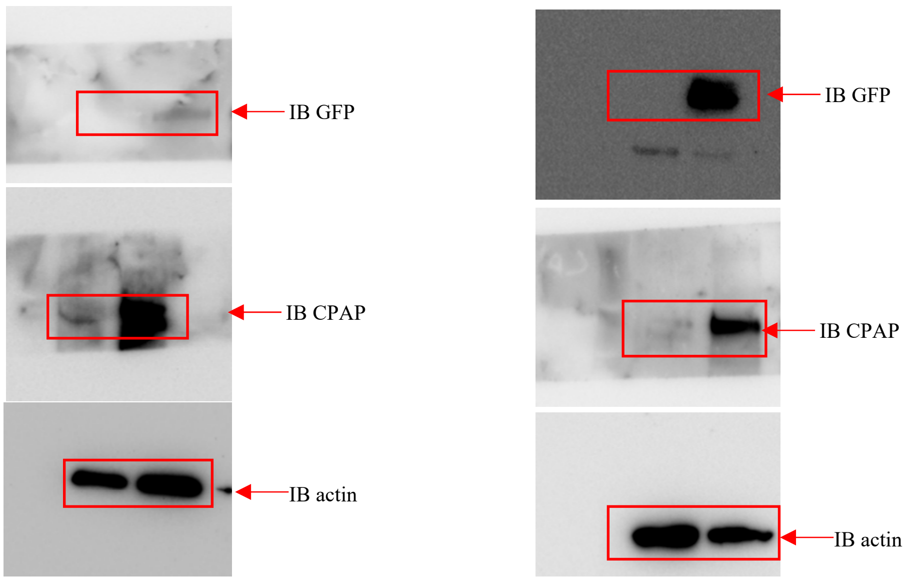

Supplemental Fig. 2B

HEK293T

HeLa

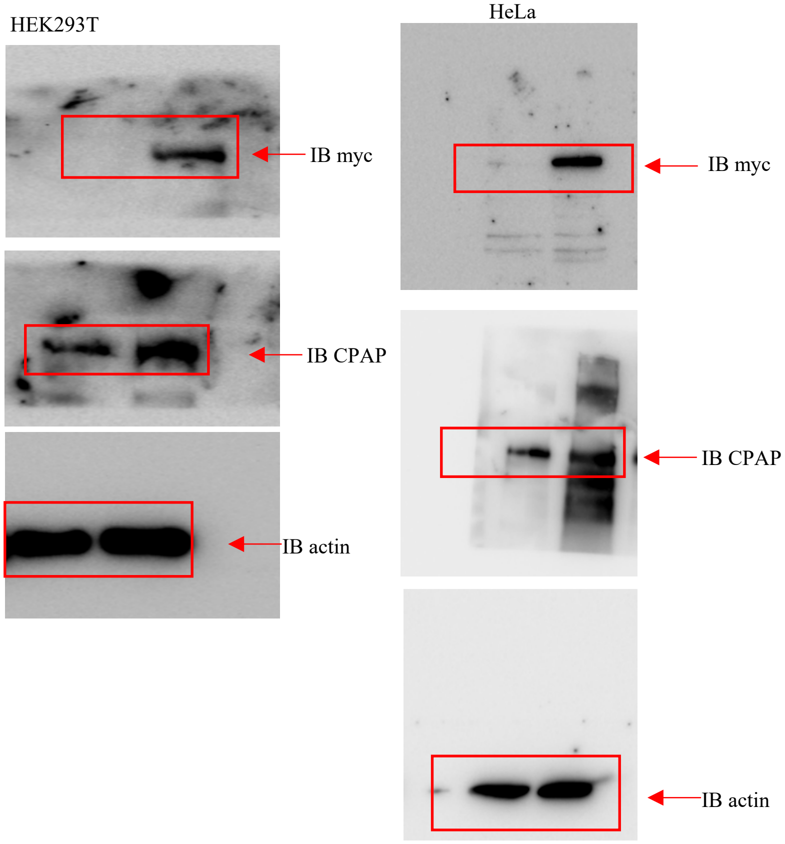

Supplemental Fig. 2C

HEK293T

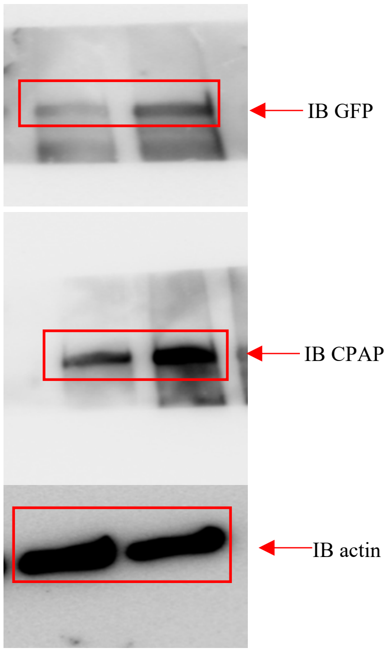

Continue supplemental Fig. 12: Original IB (scanned x-ray film or ChemiDoc chemiluminesence imager) images. Please note that, for many of the IBs, original WB membranes were cut based on the molecular weight markings before probing using different antibodies. Hence, many comparisons were done using data generated using the same blot, but no parallel blots.

Supplemental Fig. 3A

HEK293T

HeLa

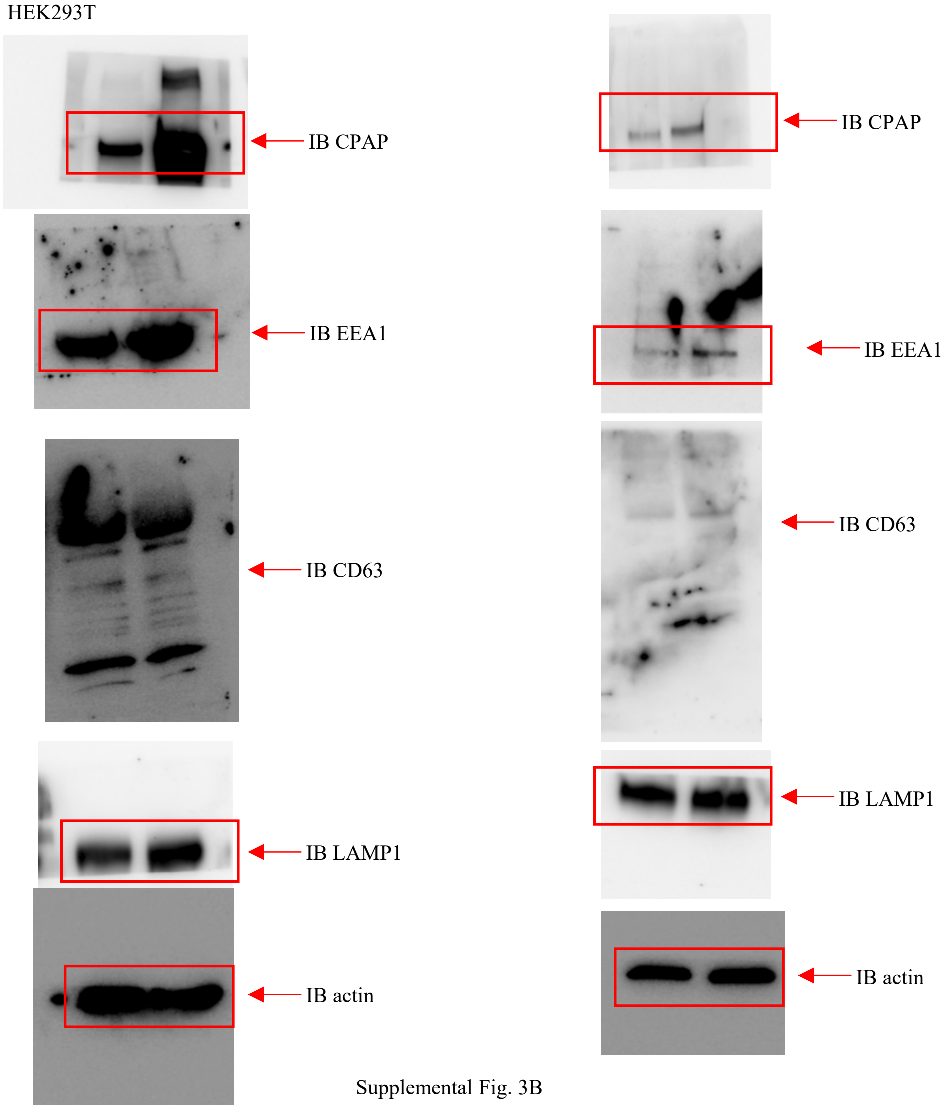

Supplemental Fig. 3B

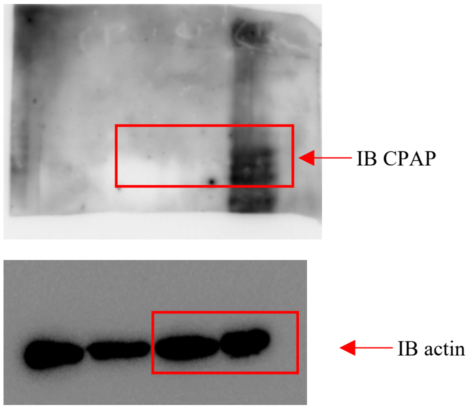

Continue supplemental Fig. 12: Original IB (scanned x-ray film or ChemiDoc chemiluminesence imager) images. Please note that, for many of the IBs, original WB membranes were cut based on the molecular weight markings before probing using different antibodies. Hence, many comparisons were done using data generated using the same blot, but no parallel blots.
